# Tryptophan, an important link in regulating the complex network of skin immunology response in atopic dermatitis

**DOI:** 10.3389/fimmu.2023.1300378

**Published:** 2024-01-22

**Authors:** Yaxin Huang, Lingna Chen, Fuming Liu, Xia Xiong, Yongliang Ouyang, Yongqiong Deng

**Affiliations:** ^1^ Department of Dermatology & Sexually Transmitted Disease (STD), the Affiliated Hospital of Southwest Medical University, Luzhou, Sichuan, China; ^2^ Department of Dermatology & Sexually Transmitted Disease (STD), Chengdu First People’s Hospital, Chengdu, Sichuan, China; ^3^ Health Management Center, Luzhou People’s Hospital, Luzhou, China

**Keywords:** atopic dermatitis, tryptophan, gut microbiota, immunology response, inflammation

## Abstract

Atopic dermatitis (AD) is a common chronic relapsing inflammatory skin disease, of which the pathogenesis is a complex interplay between genetics and environment. Although the exact mechanisms of the disease pathogenesis remain unclear, the immune dysregulation primarily involving the Th2 inflammatory pathway and accompanied with an imbalance of multiple immune cells is considered as one of the critical etiologies of AD. Tryptophan metabolism has long been firmly established as a key regulator of immune cells and then affect the occurrence and development of many immune and inflammatory diseases. But the relationship between tryptophan metabolism and the pathogenesis of AD has not been profoundly discussed throughout the literatures. Therefore, this review is conducted to discuss the relationship between tryptophan metabolism and the complex network of skin inflammatory response in AD, which is important to elucidate its complex pathophysiological mechanisms, and then lead to the development of new therapeutic strategies and drugs for the treatment of this frequently relapsing disease.

## 1 Atopic dermatitis

Atopic dermatitis (AD) is a chronic inflammatory skin disease that results from a complex interplay of genetics, environment, and immunity, which is characterized by intense itching, recurrent eczema lesions, and a personal or family history of atopy (1). The incidence of AD is gradually increasing with the development of the current industrialization and urbanization, affecting up to 15-30% of children and 10% of adults worldwide (2, 3). Even worse, approximately 50-70% of children with AD are at risk of developing other atopic diseases such as allergic asthma and/or allergic rhinitis in the future (4). At present, researchers believe that immune disorders play a crucial role in the pathogenesis of AD. T helper type 2 (Th2) and Th17-mediated immune disorders dominates the acute stage and chronic stages of AD respectively (5), both of them “interweave” each other to form a complex network of skin inflammation. Among them, the Th2 type immune response has attracted widespread attention and exploration. In relevant studies on the pathogenesis of AD, the pathogenic role of keratinocyte derived cytokines such as thymic stromal lymphopoietin (TSLP), interleukin-33 (IL-33) and IL-25 in inducing skin inflammation has been emphasized (6). These cytokines can promote the production of Th2 type cytokines such as IL-4, IL-5, and IL-13. Notably, IL-4 and IL-13 have previously been reported to damage the integrity of the skin barrier by inhibiting the production of key proteins such as filaggrin (Flg) and disrupting the stability of tight junctions (7, 8), leading to increased penetration of allergens and pathogens. The downstream signal transduction of both also blocks the expression of innate immune response genes such as β-defensins (7, 9), increasing the risk of skin infection with *Staphylococcus aureus* in AD patients. Meanwhile, IL-4 and IL-13 can drive the regeneration of eosinophils and mast cells, as well as stimulate the secretion of key cytokines IL-31 in pruritus stimulation (10). The repeated scratching behavior leads to further physical damage to the skin barrier, thereby forming a “vicious cycle” that exacerbates AD skin lesions. In addition, congenital skin barrier dysfunction and disruption of the microbiota in the skin and intestines are also considered the main contributors in driving the development of AD (11). However, the exact mechanism of AD pathogenesis mediated by intestinal microbiota is still not fully elucidated, which is presumed to be associated with tryptophan (Trp) metabolism.

Long-term itching symptoms and recurrent episodes of disease could severely affect the life quality of AD patients, and are closely related to the occurrence of negative psychology such as anxiety and depression (12). But, unfortunately, treating AD is challenging due to the high heterogeneity of the disease (13) and the limited therapeutic drugs (14). In traditional treatment regimens, topical corticosteroids are a classic first-line medication, although they could produce anti-inflammatory and immunosuppressive effects by inhibiting various inflammatory cells and cytokines, long term use may be limited by the side effects such as skin atrophy (15). The highly individualized selection of immunosuppressive drugs reduces the scope of application of the drugs and patient compliance. As an emerging treatment for AD, biological agents seem to be effective alternatives to traditional treatment. In 2017, dupilumab, a fully monoclonal antibody targeting IL-4R, became the first biological agent approved for use in adult patients in the United States due to its significant improvement in AD clinical manifestations. However, a higher incidence of herpes simplex virus infection and conjunctivitis was found in patients receiving dupilumab. Among other newly developed monoclonal antibodies, including Lebrikizumab and Tralokinumab combined with IL-13, Nemolizumab inhibiting IL-31, and Fezakinumab blocking the action of IL-22, various degrees of herpes infection, conjunctivitis, viral upper respiratory tract infection, peripheral edema, and elevated creatinine kinase levels have been found after treatment (16). Recent studies have shown that the activation of the JAK (Janus kinase)- STAT (signal transducer and activator of transcription) signaling pathway is essential for mediating downstream inflammatory cytokines in AD patients, including IL-4, IL-5, IL-13, IL-31, IL-22, and TSLP (17). These cytokines bind to immune cells, keratinocytes, and peripheral sensory neurons, leading to the spread of inflammation and itching symptoms in AD patients. Therefore, JAK inhibitors have become a new treatment strategy for AD because of immunosuppressive effects. Currently, commonly used JAK inhibitors include oral Baricinib targeting JAK1 and 2, and Upadacitinib targeting JAK1, as well as topical Tofacitinib targeting JAK1 and 3, Ruxolitinib targeting JAK1 and 2, and Delgocitinib targeting the entire JAK pathway. However, it is worth noting that, although JAK inhibitors could improve the severity of the condition and itching symptoms in AD patients, they also have the risk of increasing blood creatine phosphokinase and inducing headaches and nasopharyngitis (18). The long-term effectiveness and safety of these developing biological therapies are not yet fully understood, and with the continuous emergence of new therapies for AD, the comparison of drugs is crucial for patients to choose safe and effective treatment plans. Hence, in-depth study of the mechanism and looking for new potential treatment of AD is still valuable. Trp metabolism has recently been found to have a potential connection with the regulation of AD immune system, and greatly affect the development of other immune diseases such as metabolic syndrome, neuropsychiatric disorders and inflammatory bowel disease (19). However, throughout the literatures, the role of tryptophan metabolism in the pathogenesis of AD has not been well discussed. This article reviews the potential role of tryptophan metabolism in regulating the complex network of skin inflammatory response in AD, in order to enhance understanding of the occurrence and development of AD and seek treatment opportunities targeting tryptophan metabolism.

## 2 Tryptophan metabolism and its’ role on regulating skin diseases by aryl hydrocarbon receptor

**Figure 1:**
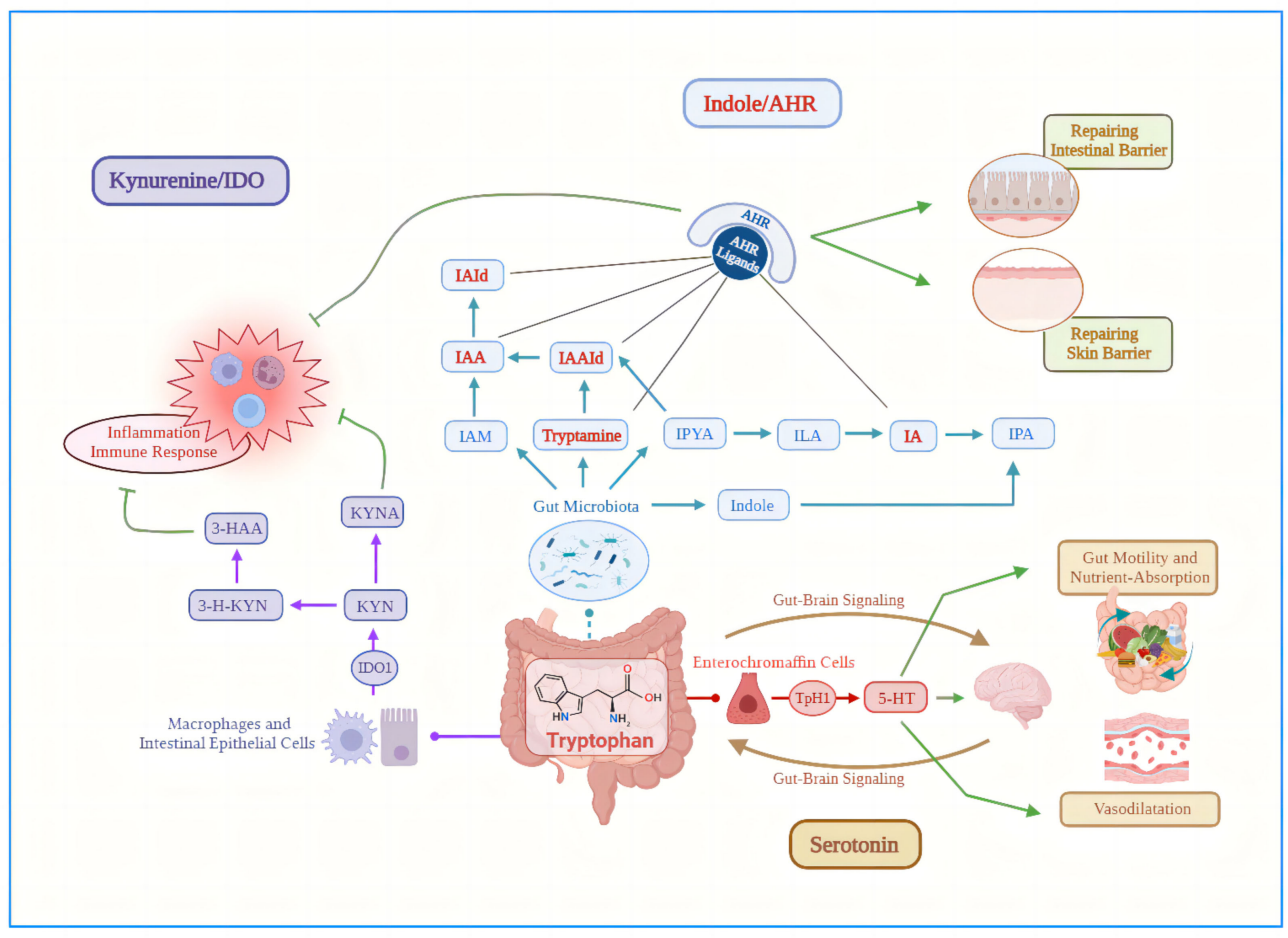
Three main pathways of tryptophan metabolism in the intestine under steady-state conditions. (i) Purple arrows: the Kynurenine pathway via indoleamine 2,3-dioxygenase 1 in macrophages and intestinal epithelial cells; (ii) Blue arrows: the direct conversion of Trp into indole, indole derivatives, and tryptamine by the intestinal microbiota via the enzyme tryptophanase; (iii) Red arrows: the serotonin pathway via Trp hydroxylase 1 in enterochromaffin cells. Green lines, inhibitory action; Green arrows, promotional effect; AHR, aryl hydrocarbon receptor; IA, indoleacrylic acid; IAA, indole acetic acid; IAAId, indole‐3‐acetaldehyde; IAId, indole‐3‐aldehyde; IAM, indole-3-acetamide; IDO1, indoleamine 2,3-dioxygenase 1; ILA, indole-3-lactic acid; IPA, indole propionic acid; IPYA, Indole pyruvic acid; KYN, Kynurenine; KYNA, kynurenic acid; TpH1, Trp hydroxylase 1; 3-HAA, 3-Hydroxy-Anthranilic acid; 3-H-KYN, 3-Hydroxy-Kynurenine; 5-HT, 5-hydroxytryptamine.

Tryptophan is an essential amino acid for the human body that is mainly produced by high-protein foods such as milk, seafood, grains and peanuts, which is a biosynthetic precursor of a large number of metabolites (19). As an important intestinal metabolite, Trp metabolism in the intestine follows three main pathways (20) under homeostatic conditions: (i) the Kynurenine (Kyn) pathway (KP) via indoleamine 2,3-dioxygenase 1 (IDO1) in immune cells (mainly macrophages) and intestinal epithelial cells (IEC); (ii) the serotonin pathway via Trp hydroxylase 1 (TpH1) in enterochromaffin cells. Above two pathways mainly occur in host cells, that are predominantly but not exclusively used by the host. (iii) The direct conversion of Trp into indole, indole derivatives, and tryptamine by the intestinal microbiota via the enzyme tryptophanase (21), which produces metabolites such as indole acetic acid (IAA), indole‐3‐acetaldehyde (IAAld), indole‐3‐aldehyde (IAld), indole propionic acid (IPA) and indoleacrylic acid (IA) (**Figure 1**). Trp metabolites mainly exists in human feces and have been shown to maintain the integrity of the skin and intestinal barrier and immune cell homeostasis by activating aryl hydrocarbon receptor (AhR) (22, 23), thus are considered as the active biomarkers (24).

AhR is a ligand dependent transcription factor that could be observed in all skin cells. Although the expression levels vary in specific cell types (25), AhR signaling plays a major role in skin diseases due to its involvement in many important physiology processes such as regulating immune cells, maintaining redox balance in cells and epidermal barrier function (25–27). As a hybrid receptor, AhR could be activated by a variety of exogenous and endogenous ligands (28–31), among them, the mild and transient AhR activation caused by antioxidant phytochemicals or some tryptophan derivatives could effectively maintain healthy and complete skin barrier (32, 33). When tryptophan derivative binds to the AhR ligand to form a complex, AhR dissociates from the cytoplasm and translocates to the nucleus. The AhR nuclear translocation protein (ARNT) dimerizes with the exposed nuclear translocation site of AhR, binding to be the upregulated target gene transcription response element (34). This signaling pathway initiates the activation of the OVO-like 1 (OVOL 1) transcription factor, and subsequently enhances the expression of Flg and olein (LOR), these kind of terminal differentiation proteins have specificity for fully differentiated keratinocytes (35), helping to accelerate the final differentiation of the epidermis and the formation of the epidermal barrier (36, 37). Notably, increasing evidences suggest that the binding of tryptophan derivatives with AhR play a role in the pathogenesis or treatment of many skin diseases (38, 39), including inflammatory diseases, skin pigmentation diseases, and cancer (40, 41). As expected, tryptophan metabolism has been reported to be closely related to the occurrence and development of AD (42), the tryptophan metabolite in the AD skin lesions is significantly reduced (43) that may be associated with the weakened tryptophan metabolism in the skin microbiota by Th2 type immune response (43). Due to AhR lacking physiological ligands in the Th2-deviated environment of AD, the skin barrier damage and colonization of pathogenic microorganisms such as *Staphylococcus aureus* would increase, thereby exacerbating Th2 type inflammation in the lesion of AD (44, 45). Therefore, we speculate that tryptophan and its derivatives could be beneficial for the treatment of AD by appropriately activating the AhR/ARNT axis.

Tryptophan has recently been also implicated in the crosstalk between gut microbiota (GM) and host in healthy and diseased states (19, 46), and its metabolic impairment may affect the occurrence and development of many diseases, such as metabolic syndrome, neuropsychiatric disorders and inflammatory bowel disease (19). Research findings showed that, compared with the healthy control, inflammatory bowel disease (IBD) patients experienced intestinal microbiota homeostasis imbalance, while their serum tryptophan levels also significantly decreased. Interestingly, the composition of GM is significantly correlated with serum level of tryptophan, dietary supplementation with Trp can normalize the disordered GM in the IBD mouse model (47), and the AhR ligands produced subsequently by GM could alleviate the inflammatory response (48). Wilck et al. found that, *Lactobacillus murinus* and *Lactobacillus reuteri* could convert tryptophan into IAld and indole-3-lactic acid (ILA) by aromatic amino acid aminotransferase and indole lactic acid dehydrogenase (49, 50), these generator of AhR would improve the severity of colitis in mice (23). This type of approach can even be expanded to other inflammatory diseases (51), suggesting that the biological effects of Trp metabolites may be related to the interactions between gut microbiota and may become potential therapeutic targets for certain diseases. Additionally, Metghalchi et al. found that plasma level of Trp predicts the likelihood of adverse cardiovascular events in patients with acute myocardial infarction (52). Based on the characteristics of Trp metabolism influenced by pathological conditions, the use of Trp and its metabolites as biomarkers to support diagnosis and prognosis and to guide treatment options is attractive. Therefore, this article will focus on exploring the potential diagnostic and therapeutic role of tryptophan metabolism in the complex pathogenesis of AD.

## 3 The potential role of tryptophan metabolism in atopic diseases

AD typically begins in early childhood and is usually the first manifestation of atopic progression. While clinical symptoms of some AD children would disappear with age, some children may experience food allergy during the course of the disease, even half of AD children may develop allergic asthma and two-thirds of AD children are at risk of developing allergic rhinitis in the future. This kind of disease progression is known as the “Atopic March” (53, 54). Exploring the relationship between these atopic diseases and tryptophan can help us further understand its role in AD.

### 3.1 Food allergy

Although people often refer to any adverse reaction to food as an allergy, strictly speaking, food allergy (FA) is an adverse reaction to the food itself that is mediated by specific IgE antibody. Increased levels of tryptophan and indole metabolic pathway products were found in serum samples from FA children, while levels of metabolites from Kynurenine and 5-hydroxytryptamine pathways decreased with disease burden (55, 56). However, these tryptophan metabolites can downregulate the T cells activity or induce various regulatory T cell (Treg) cells to regulate immune responses at the mucosal barrier (55, 57). The reduction of tryptophan metabolites with potential anti-inflammatory effects would create a suitable environment for Th2 tilted immune responses, thereby increasing the occurrence risk of Th2 related diseases such as AD. Another study on peanut allergic mice suggests that the above mechanism is related to the activation of AhR (58, 59), but to our knowledge, there are currently no clinical studies evaluating the functional role of AhR ligands in FA.

### 3.2 Allergic rhinitis and asthma

Atopic diseases are highly prevalent in children worldwide, with the prevalence rates of allergic rhinitis (60, 61) and asthma (62) being approximately 14% and 15%, respectively. Consistent with food allergy, levels of tryptophan are significantly elevated in patients with allergic rhinitis and asthma (55, 63–66). Serum tryptophan levels could not only be used to distinguish between stable asthma children and healthy children, but also to distinguish between controlled and uncontrolled asthma patients (64). This kind of influence could even be traced back to the early stages of life, the tryptophan metabolite 5-hydroxyindolepyruvate in maternal amniotic fluid can determine whether the baby has wheezing attacks in the first year of life (65). The above evidences suggest the important value of tryptophan in atopic related diseases. Some researchers believe that, the high level of tryptophan in the serum of patients with atopic diseases may be related to the low activity of IDO-1 enzyme or the inhibition of IDO-2 enzyme activity (66, 67). Among them, IDO-1 is widely expressed in tumor cells and inflammatory/antigen presenting cells (APCs), induces the production of Kyn by catabolizing tryptophan, the ratio of serum Kyn to tryptophan reflects IDO-1 activity to some extent (68, 69). Kyn could up-regulate the expression of foxp3 transcription factor, increase the differentiation of Treg cells and decrease the expansion of Th17 type cells (67, 70–72). For instance, Th17 and Th1 type airway inflammation were observed in IDO deficient mice infected with the virus, characterized by significant infiltration of neutrophil, high production of IL-17 and interferon-γ (IFN-γ) and obvious airway responsiveness (73). In the asthma mouse model, activation of toll-like receptors (TLR)-9 through bacterial DNA motifs could induce the expression of IDO (74) and aromatic receptor (75, 76) to reduce the inflammatory airway hyperresponsiveness. Therefore, although the specific role of tryptophan metabolism in the pathogenesis of atopic diseases such as asthma and allergic rhinitis is still not fully understood, it cannot be denied that tryptophan metabolism and its intermediate products are the important links in regulating immune responses (19). According to research reports, administration of D-tryptophan before experimental asthma induction in mice observed an increased number of regulatory T cells in the lungs, a decreased Th2 type immune response, and an improvement in allergic airway inflammation and airway hyperresponsiveness (77). Another tryptophan metabolite, 3-Hydroxy-Anthranilic acid (3-HAA), could directly target the phosphorylation of 3-phosphoinositol dependent kinase 1 (PDK 1) to inhibit NF-κB triggered by T cell antigen receptors, leads to dysfunction and cell death of activated TH2 cells in the body, which is sufficient to suppress experimental asthma induced by TH2 immune response in mice (78). In addition, PapaMichael et al. (79) also reported a positive correlation between the tryptophan metabolite 5-hydroxyindoleacetic acid (5-HIAA) and the asthma FEV 1/FVC (forced expiratory volume in the first second/forced vital capacity) ratio, as well as a negative correlation with fractional exhaled nitric oxide (FeNO). However, further research is needed to demonstrate the therapeutic potential of tryptophan and its metabolites in patients.

In summary, the atopic diseases such as food allergy, asthma, and allergic rhinitis all have varying degrees of tryptophan metabolism abnormalities, which may even affect early immune homeostasis. The use of tryptophan metabolites as biomarkers to support the diagnosis and prognosis of atopic diseases and guide treatment choices is an attractive choice. However, despite the extensive research supporting the important role of tryptophan metabolism in the occurrence and development of atopic related diseases, the mechanism connections between these diseases are still unclear (80), the specific mechanisms of action and therapeutic potential still need to be further explored.

## 4 Trp metabolism as a bridge between atopic dermatitis and gut microbiota

### 4.1 Gut microbiota and its metabolites contribute to the development and prognosis of AD

GM is an enormous and complex ecosystem. The number of bacterial cells within the human gut outnumber the host’s cells by 10 times and the genes encoded by these bacteria even exceeds their host’s genes by more than 100 times (81). Consequently, extensive research has been conducted on the gut microbiota and its role in the various diseases and health states. It is reported that the imbalance in the diversity and composition of GM could lead to negative changes in intestinal microbial metabolism and immune response, such as affecting the host’s intestinal immune environment and barrier function, disrupting mucosal immune tolerance, and increasing the vulnerability of the host (82, 83). Actually, the connection between GM and skin inflammation was discovered as early as the 1930s (84). Up to now, numerous studies have revealed that the development of allergic diseases such as asthma, allergic rhinitis and AD is closely associated with GM disturbance (85–91). Although there are some conflicting results (92–95), lots of clinical trials have claimed that AD patients exhibit poor gut microbial diversity compared to healthy individuals (96–99), as well as a structural disorder in the gut microbiota, which includes the increased abundance of microorganisms related to inflammation and epithelial damage in the intestinal flora, such as *Clostridium difficile*, *Coliform*, pathogenic *Escherichia coli*, and *Staphylococcus aureus (* 93, 97, 100–104), and the significantly decreased abundance of SCFA (short chain fatty acid) producers, such as *Bifidobacteria*, *Fecal cocci* and *Fecal bacilli* (92, 95, 96, 103, 105–108). Notably, SCFAs, including acetic acid, propionic acid, and butyric acid, have been proven to help maintain the balance of GM, affect immune cells, and are closely related to the remission of clinical manifestations of AD (96, 109, 110). SCFAs can bind G-Protein-Coupled Receptors (GPCRs, including GPR43, GPR41, and GPR109a) expressed on intestinal epithelial and immune cells to inhibit histone deacetylase (HDAC) (111, 112), leading to acetylation of the Foxp3 coding region in T cells, driving Treg differentiation (113, 114), downregulating expression of inflammatory cytokines (IL-6, IL-8, and tumor necrosis factor -α (TNF-α)) (115, 116), and stimulating the production of anti-inflammatory cytokines such as IL-10 by peripheral blood mononuclear cells (PBMCs) (117) which could suppresses pro-inflammatory types of Th17 and Th2 cells in turn. Therefore, intervention targeted GM and its metabolites may be an alternative method for controlling the inflammationary responses and improving the clinical symptoms of AD.

### 4.2 Probiotics alleviate the inflammatory response in AD by up-regulating tryptophan metabolism

In addition to SCFA, another prominent example of how microbiota affects host tissue level immune maturation is the probiotic metabolic pathway of tryptophan. As the important regulator of GM, probiotics could improve the clinical severity of patients by the reduction of pro-inflammatory cytokines such as IL-13 and IL-5 in AD skin lesions (118). It has been revealed that IL-13 is a key driver of activating Th2 type immune responses, and IL-15 is a key cytokine inducing development and survival of eosinophil (119). Thus, probiotics is expected to become an effective alternative strategy for the treatment of skin diseases based on the enormous potential in regulating immune function (120, 121). It is worth mentioning that, current research has also confirmed a close link between probiotics and tryptophan metabolism. For instance, the application of *Lactobacillus reuteri* (122), *Lactobacillus salivary* (123), and *Bifidobacterium* (124) significantly increased the level of tryptophan metabolites in the serum, and even accompanied by the decreased pro-inflammatory response. In fact, tryptophan, as an important regulator of mammalian inflammatory response (125), has been proven to have an immunomodulatory effect in experimental colitis or IBD patients (126). The GM obtained from IBD patients showed poor ability to produce AhR ligands (23), and in addition, sterile mice susceptible to colitis observed significant improvement in intestinal inflammation after supplementation with AhR agonists and *Lactobacillus* strains capable of metabolizing tryptophan (22). Similarly, administration of *Bifidobacterium* in AD model mice showed reduced scratching behavior and an increase in the level of Kyn (127). Therefore, considering the role of the beneficial microbiomes in tryptophan metabolism, we speculate that increasing intestinal probiotics may medicate the immune inflammatory responses and alleviate clinical symptoms of AD by producing tryptophan derivatives.

### 4.3 Tryptophan metabolites promote the regression of skin inflammation in AD by repairing the intestinal barrier

It has been reported that, Trp metabolism and its derivatives have many beneficial effects on intestinal epithelial barrier function mediated by GM. The intestinal barrier is made up of epithelial cells, mucous layers, T cells, IgA, and dendritic cells, collectively forming the “mucosal firewall” (128). The imbalance of GM, decreased production of SCFA, and loss of immune tolerance in the intestines of AD results in the occurrence of inflammatory reactions, increased pathogenic microorganisms and damaged intestinal barrier (92). This kind of “leaky gut” paves the way for the occurrence of AD skin inflammation by releasing toxins, food residues and pathogenic microorganisms from the damaged intestinal epithelium into the systemic circulation, inducing the release of pro-inflammatory cytokines to propel Th2 type immune responses ultimately (129–131). Nevertheless, Trp metabolites such as indole can reinforce intestinal epithelial barrier function by enhancing the expression of genes involved in preserving epithelial cell structure and function *in vivo* and *in vitro* (132, 133), IA (134) and IPA (134, 135) can enhance intestinal epithelial barrier function by reducing the expression of inflammatory factors in intestinal epithelial cells via activating the AhR and PXR (pregnenolone X receptor) receptors, respectively, to promote intestinal goblet cells differentiation and mucus production. Thus, it is supposed that the supplementation of tryptophan metabolites will play a beneficial role in AD through repairing the intestinal epithelial barrier function and consequently controlling “leaky gut” induced skin inflammation.

## 5 Tryptophan metabolites regulate the complex immune response in AD

Although the exact mechanisms of the disease pathogenesis remain unclear, accumulating evidence from experimental, genetic, and clinical studies indicates that impaired skin barrier and the immune dysregulation are the critical etiologies of AD (11). Although AD has long been considered to be Th2-dominated inflammation, it is also evident that its pathology is accompanied by an imbalance in immunity involving both innate immune cells such as keratinocytes, macrophages, dendritic cells, and adaptive immune cells such as Th1, Th2, Th17, Th22 and Treg cells, which interact and eventually intertwine into a complex inflammatory network. On the other hand, tryptophan metabolism has been firmly established as a key regulator of both innate and adaptive immune cells (68, 136), and its derivative receptor, AhR, is a key component of the immune response at the barrier site. Alterations in AhR activity or AhR deficiency may disrupt the immune response or impair the development and function of the epidermal barrier (137). Next, we will delve into the innate and adaptive immune mechanisms mediated by the tryptophan metabolites in the pathological process of AD.

### 5.1 Keratinocytes

Keratinocytes represent the first line of the host defense system by sensing pathogens via innate immune receptors, initiating antimicrobial responses and producing various cytokines and antimicrobial peptides. Among the dysregulation of immune responses in AD, keratinocytes initiate cross-talk between innate and adaptive immune responses by regulating the release of several key molecules including eotaxin/CC chemokine ligand (CCL) 11, eotaxin‐3/CCL26, monocyte chemotactic protein (MCP)‐4/CCL13, regulated on activation, normal T cell‐expressed and secreted (RANTES)/CCL5, and thymus and activation‐regulated chemokine (TARC)/CCL17, that trigger inflammatory reactions and immune responses (138). It was found that Th2 type cytokine IL-4/IL-13 could stimulate the production of IL-24 in Keratinocyte by inducing the activity of STAT 6 (139), and then reduce the expression of Flg, ultimately leading to epidermal terminal differentiation damage and barrier dysfunction in AD patients. Interestingly, AhR ligands, such as Coal tar, Glyteer and 6-formylindolo[3,2-b]carbazole (FICZ), could block the activation of STAT 6 mediated by IL-4/IL-13 and promote the expression of Flg via mild and transient activation of AhR/ARNT pathway, thus restoring barrier dysfunction (140, 141). Further experiments have proved that the defect of AhR in Keratinocyte may be the cause of worsening inflammation (142), while intense and sustained AhR activation would lead to excessive keratinization of Keratinocyte and sebocytes (43). In conclusion, the lack of AhR or the change of its activity is related to the imbalance of Keratinocyte ‘response to inflammatory stimuli, which may damage the immune response or the normal function of the epidermal barrier.

### 5.2 Macrophages

Macrophages (Mφ), as a natural immune cell, differentiate into two phenotypes, including the M1 phenotype activated by TLR ligands to secrete proinflammatory cytokines such as TNF-α and IL-1β, or the M2 phenotype activated by interferon or lipopolysaccharide (LPS) to secrete the anti-inflammatory cytokine such as IL-10 (143), and respond to immune responses in the AD microenvironment under different activation states. Remarkably, M2 macrophages exert immunosuppressive effects by expressing IDO to depresses T cell proliferation and stimulate regulatory T cells, which are cells with immune suppressive function (144, 145). Tryptophan metabolism has been found to affect the polarization and immune function regulation of macrophages. 5-methoxyltryptophan (5-MTP), a new endothelial factor produced by L-tryptophan metabolism that has recently been identified as a functional feature with protective and repair barriers (146), could inhibit the LPS-induced p38-MAPK pathway by interfering the binding of phosphor-p38 to peroxiredoxins (Prdx), thereby blocking the activation of macrophages and preventing the occurrence of systemic inflammation. Similarly, for other tryptophan metabolites, I3A could reduce the expression of inflammatory cytokine IL-1qwβ, TNF-α and CXCL-1 in macrophages (146); IA could stimulate IL-10 production in macrophages, thereby reducing the secretion of TNF and IL-6 (115); Kyna was later confirmed to reduce the inflammatory response induced by LPS stimulation in monocytes and Mφ through its interaction with GPR35 (147). Therefore, these findings support the idea that tryptophan metabolism could effectively control macrophage activation and “cytokine storm” *in vivo*, but the specific mechanism of action in AD needs further research.

### 5.3 Dendritic cells

As early as the 1970s, dendriticcells (DCs) has been known to coordinate immune responses by building a “bridge” between innate and adaptive immunity, while the normal physiological function of DCs could be severely affected under pathological condition, and results in the occurrence of abnormal immune responses subsequently. For instance, there are various inflammatory signals in the AD microenvironment, exposure of DCs to this immune stimulation environment would upregulate the expression of inflammatory mediators during antigen acquisition, and activate effector T cells via treated antigen peptides simultaneously (148). Despite not being the case for all DCs (149), exposure to stimuli such as IFN, TLR-4, TLR-9, TNF, and IL-1 could induce functional IDO expression in DCs, promoting tolerance *in vivo* and contributing to their ability to present antigens and stimulate T cells (150) either through effects of IDO on DCs or through direct action on T cells mediated by tryptophan depletion or tryptophan metabolites (151, 152). A previous study proposed initially that CD4+naïve treated T cells could transformed into FoxP 3+functional regulatory T cells upon exposure to LT/Kynurenine or IDO+ DCs (153). Additionally, the tryptophan metabolite IPA promotes the accumulation of anti-inflammatory DCs in the mesenteric lymph nodes, which was abolished by AhR antagonist (154). IPYA inhibits colon inflammation by increasing IL-10 production, reducing Th1 cell differentiation in the lamina propria of the colon, and altering the composition of DCs in the mesenteric lymph nodes (154). Therefore, we believe that the tryptophan metabolism mediated by DCs has a positive inflammatory regulatory potential in AD.

### 5.4 Th2 cells

The strong activation of adaptive immunity driven by Th2 cells seems to be the dominant mechanism in the acute phase of AD (155), mainly characterized by the secretion of cytokines such as IL-4, IL-5, IL-13 and IL-31, which are involved in the occurrence of keratinocyte apoptosis, inflammation and itching symptoms, to confirm the influence of T cells in the pathogenesis of AD. Previous studies have shown that tryptophan and its derivative ligand, AhR, are involved in regulating T cell populations, mediating immunosuppression, and maintaining the balance between Treg and T cells. A recent study has demonstrated that Th2-deviated environment is related to the reduction of the endogenous AhR ligand such as indole-3-aldehyde (IAId) produced by the skin symbiotic microbiota (43). IAId could promote the interaction of activated AhR receptors with TSLP, thereby inhibit the production of TSLP, and ultimately reduce the occurrence of AD-like dermatitis (43). Notably, TSLP, mainly produced by epidermal keratinocytes and fibroblasts, could stimulate Th2 differentiation by promoting the migration of DCs to the epidermis (156), playing a central role in initiating Th2 type adaptive immune responses in AD skin inflammation. In addition to IAId, significant alleviation in clinical manifestations and reversal of Th2 biased immune response were also observed after supplementation with D-tryptophan in a mice with allergic airway inflammation (77). D-tryptophan is a metabolite of *Bifidobacteria*, *Lactobacillus* and *Lactococcus*, strongly induces the production of anti-inflammatory factor IL-10 and reduces the secretion of IFN‐γ,IL‐12, and IL‐5 in LPS‐induced KM‐H2 (a human Hodgkin’s disease cell line) cultures, and suppress the expression of the crucial chemokine CCL17 responsible for recruitment of Th2 cells in AD skin lesions (77, 141). A newly‐identified role of D‐tryptophan consists of slowing down the production of biofilm formation in *Staphylococcus aureus* and *Pseudomonas aeruginosa (* 157, 158). As is known, the skin of AD patients is more susceptible to the colonization and overgrowth of *Staphylococcus aureus* (159), which has been linked to the increased IgE responses and severity of AD disease (160). In addition, Yu et al. found that appropriate activation of the AhR/ARNT/Flg axis may be beneficial in treating AD (43, 161). Th2-deviated environment could significantly reduce the expression of filaments and other barrier related molecules, while the activation of the AhR/ARNT/Flg signaling pathway by rapidly metabolized AhR ligands (such as IAId or FICZ) and dioxins (2, 162) would initiate the activation of OVOL 1 transcription factors, enhance the expression of Flg and LOR, thereby contributing to accelerating the final differentiation of the epidermis and the formation of the epidermal barrier (25, 35). It should be mentioned that, FICZ, as an endogenous UVB photoproduct (28), has been found to be closely related to human skin physiology (163) that could limit the production of IL-17 and IL-22 in mouse dermatitis models by activating the AhR receptor (142, 161), this barrier-protecting effect may partially explain why UVB phototherapy is effective in treating AD and psoriasis (164, 165). Therefore, the reduction of filaments and other barrier related molecules in the Th2-deviated environment, as well as the deficiency of AhR ligands, may underlie the skin lesions in AD (117), which may compensate for the up-regulation of AhR/ARNT signal transduction pathways to weaken the occurrence of Th2 type response mediated skin inflammation. However, the excessive activation of AhR may also induce the occurrence of pruritus dermatitis (2, 166, 167).

### 5.5 Th22 type immune response

IL-22, the effector cytokine of Th22 type immune, has been reported to play a leading role in the pathogenesis of AD. IL-22 could inhibit epidermal differentiation and promote inflammation in AD skin lesions by inducing the secretion of IL-6, and downregulate the expression of keratinocyte fibril aggregation proteins in keratinocytes, especially, to increase the degree of epidermal damage (168), which is closely related to the severity of AD. However, the role of IL-22 in AD pathogenesis may be bidirectional, as it has been reported to have protective effects in acute viral infection of the intestinal tract but is pathogenic in the chronic inflammatory environment of AD and rheumatoid arthritis (169–172), this contradictory characteristic can even be derived into tryptophan metabolism. For instance, topical application of the tryptophan metabolic derivative FICZ could reduce the gene expression of IL-22 in a murine mite-induced dermatitis model by activating AhR receptors (161). On the other hand, IAld and I3A could actively stimulate IL-22 secretion via AhR, and the STAT3 phosphorylation is subsequently induced to accelerate proliferation of enterocytes for restoring damaged intestinal mucosa (173). Although there is evidence linking tryptophan metabolism to IL-22 secretion, the specific role of Th22 type immune response in the pathological development of AD or its connection with tryptophan require further relevant experimental confirmation.

### 5.6 Th17 type immune response

IL-17 is an important pro-inflammatory cytokine secreted mainly by Th17 type cells (174), could results in inflammatory reactions by inducing the production of pro-inflammatory cytokines and chemokines in keratinocytes, neutrophils and endothelial cells (175, 176). Besides that, IL-17 would reduce the expression of Flg in keratinocytes (177), thereby promoting the colonization of *Staphylococcus aureus* in damaged epidermal barrier of AD. The superantigen Staphylococcin B secreted by *Staphylococcus aureus* reversely promotes the secretion of IL-17, leading to a “vicious cycle” further destroys the skin barrier function of AD (178). As was expected, tryptophan metabolites and their AhR receptors have recently been shown to control inflammation by reducing the proliferation of Th17 lymphocytes (49, 179, 180). In mice exposed to *Colitogenic* DSS, the tryptophan derivative dioxin TCDD triggered AhR to inhibit proliferation of Th17 and induce differentiation of Treg (181). Similarly, the AhR ligand 3,3’-diindolyl methane (DIM) alleviated oxazolone induced experimental colitis by reducing Th2/Th17 cells and increasing Tregs (182). Increased expression of IL-17A in CD4+T cells was also observed in AhR -/- mice (183). The above evidences suggest that, the tryptophan metabolite, as an AhR ligand, is crucial for stimulating or inhibiting host immune responses and may be involved in the innate and adaptive immune response regulation of AD, although the further research about direct relationship between probiotics/tryptophan metabolites and AD is urgently needed.

## 6 Kyn-IDO pathway plays a crucial role in the development of AD

The Kyn - IDO pathway involved in the degradation of tryptophan was initially considered to have tolerance and immunomodulatory effects, moreover, there is mounting evidence that the Kyn-IDO pathway plays a crucial role in the development of atopy and allergy Kyn and its metabolites cover more than 90% of tryptophan metabolism, have been reported to activate AhR receptors with anti-inflammatory activity (184–187), thereupon then suppressing the activity of natural killer cells (NKT) (188) and APC such as dendritic cells (DC), monocytes, and macrophages (189, 190) in mice. Therefore, the Kyn-AhR axis has been postulated to constitute one of the factors affecting the progression of chronic inflammation (184). Further research has shown that the treatment of ovalbumin (OVA) induced asthma mice with Kyn metabolite HAA could inhibit Th 2 lung inflammation through moderately inducing apoptosis of Th 2 cells (78). In addition, IDO 1-Kyn-AhR signaling may reveal feedback loops related to inflammation and reactive oxygen species (ROS) production (191, 192). In the Kyn pathway, pro-inflammatory cytokines induce the IDO1 to create more Kyn, which in turn stimulates the expression of IDO1 by activating AhR. For example, when IFN- γ acts on intestinal epithelial cells, IDO could be induced to interfere with the expression of IL-10 receptor. Subsequently, Kyn restores the upregulation of IL-10 receptor expression by activating AhR, which significantly reduces the occurrence of intestinal inflammation (193). The above evidences indicates that the anti-inflammatory properties of the Kyn pathway is achieved through a negative feedback regulatory mechanism.

IDO is widely expressed in various types of cells, including most tumor cells, dendritic cells, macrophages, microglia, eosinophils, fibroblasts, and endothelial cells (142, 191–197), the most significant and effective inducers of IDO expression mainly are cytokines (such as IFN-γ, IFN-α, IFN-β and IL-10), as well as signaling through TLRs (198–200). IDO has long been considered to contribute significantly to the control of systemic inflammation (201), including its key role in reducing Th1 cell proliferation and inducing Treg cell differentiation (151, 189, 202), which could maintain normal immunological tolerance and limit the occurrence of tissue damage in the body. In support, enhanced CD4+ Th17 and Th1 responses were observed in airway of IDO-ablated mice following attacking by OVA and infection of human rhinovirus (hRV), characterized by significant infiltration of Th 17 and Th1 type neutrophils, high production of IL-17A, and IFN- γ, and increased collagen deposition and epithelial proliferation (73). Additionally, the direct role of IDO in inhibiting target cells by activation of Treg cells has been confirmed from Munn’s recent study (203, 204), specifically, the enhanced infiltration of effector T cells in the lung cells of IDO−/− mice coincided with a sharp decrease in CD4+CD25+FoxP3+Treg cells, which could be attributed to decreased AhR activity and impaired Kyn production (185, 205). Similarly, when co cultured with IDO+AML (Acute Myeloid Leukemia) cells, naïve T cells would convert to FoxP 3+Treg cells, and yet this conversion was completely eliminated by IDO inhibitor (206). The transformed Treg cells would subsequently suppress antigen-induced T cell responses in a “time delayed manner”, so then offsetting the overactive immune response and bringing the immune system closer to physiological equilibrium. Compared to the inhibitory effect of IDO on Th 1 cells differentiation, the effect on Th 2 cells is more complex with both inhibitory and stimulatory actions reported (207). According to the report, a asthma mouse model sensitized with ovalbumin (OVA) observed that expression of IDO inhibited Th2 type airway inflammation in the lungs (74), while the IDO-expressing and Kynurenine-producing eosinophils co cultured with Th1 or Th2 cells, a preferential decrease in Th1 response and subsequent increase in Th2 cytokine production were observed (208), indicating that IDO-expressing eosinophils may create cytotoxic metabolites that maintain an imbalance between Th1 and Th2 cell populations, and the consistent results were obtained in the study of Molano et al. (209). Notably, Kositz et al. reported that the higher tryptophan levels in atopic patients may be result from lower IDO-1 activity (66). In contrast, Th2 cytokines such as IL-4 and IL-13 inhibit the expression of IDO (210, 211), which creates favorable conditions for a Th2 tilted immune environment, thereby increasing the risk of Th2 related diseases. Although the above evidences suggest that IDO and/or its metabolites have a protective effect in atopic inflammation, there are also studies demonstrating the contradictory results that expression of IDO on eosinophils may contribute to the development of allergic inflammation (195) and was up-regulated in the AD skin (212). Under these circumstances, we suspect that the upregulation of IDO expression is a result of efforts to prevent ongoing allergic inflammation. In a few words, whether the induction of IDO can inhibit the development of allergic reactions or induce immune tolerance of allergic inflammation has begun requires further research.

## 7 Discussion

**Figure 2:**
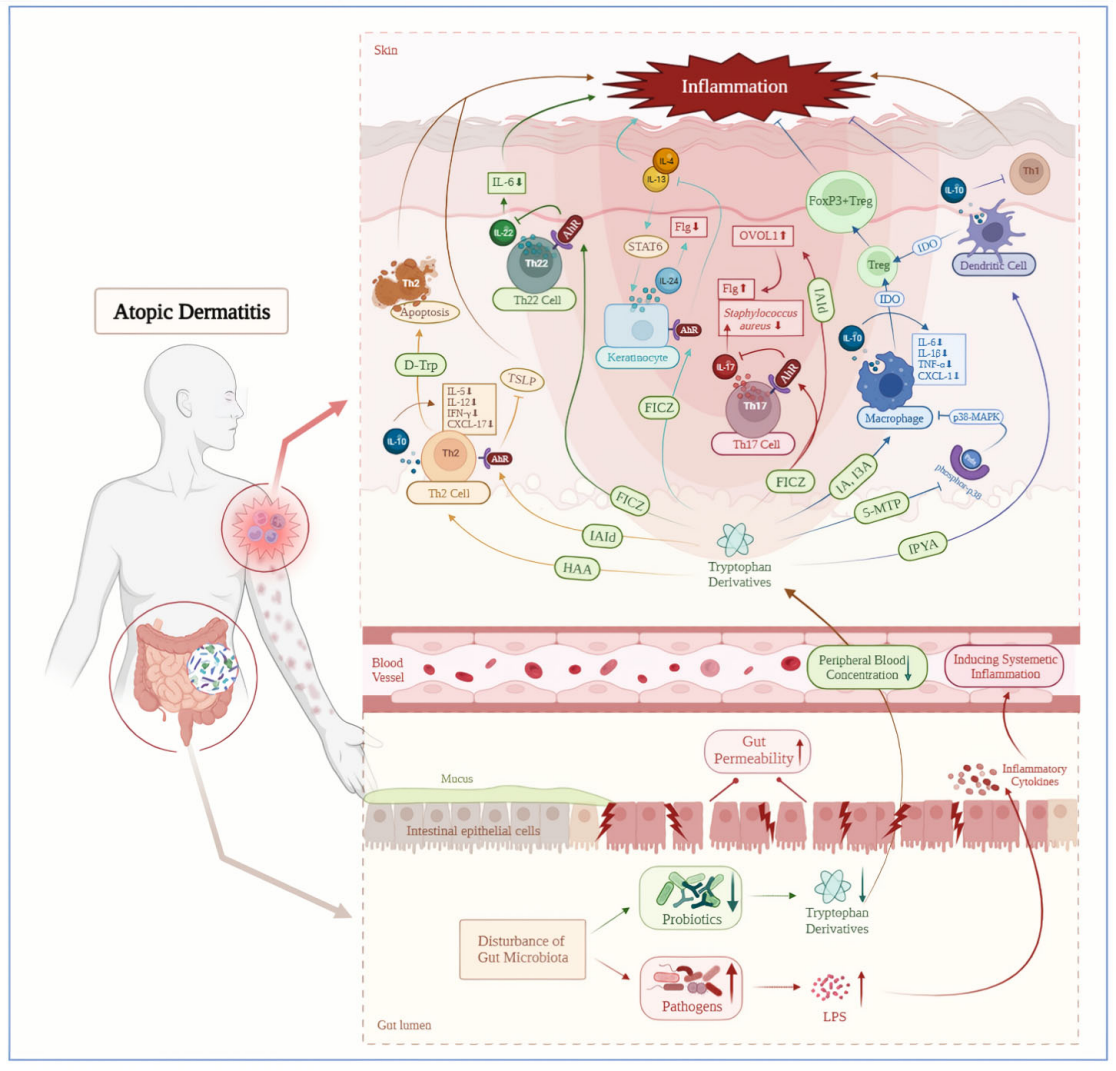
Decreased tryptophan metabolites mediated by gut microbiota disorder induce atopic dermatitis inflammation. AD patients are accompanied by intestinal microbiota disorders, including a downregulation of tryptophan producing probiotics and an increase in lipopolysaccharide producing pathogenic bacteria. The LPS could enter the bloodstream through increased gut permeability and cause systemic inflammation subsequently. On the contrary, downregulated tryptophan and its derivatives in the circulatory system have a decreased positive regulatory ability on the immune system, which could further exacerbate skin inflammation in AD patients. AhR, aryl hydrocarbon receptor; D-Trp, D-Tryptophan; FICZ, 6-formylindolo[3,2-b]carbazole; Flg, filaggrin; FoxP3+Treg, FoxP3+ regulatory T cell; HAA, Hydroxy-Anthranilic acid; I3A, Indole-3-aldehyde; IA, indoleacrylic acid; IAId, indole‐3‐aldehyde; IDO, indoleamine 2,3-dioxygenase; IPYA, Indole pyruvic acid; LPS, Lipopolysaccharide; OVOL1, OVO like transcriptional repressor 1; Prdx, peroxiredoxins; STAT6, signal transducer and activator of transcription 6; Treg, regulatory T cell; TSLP, thymic stromal lymphopoietin; 5-MTP,5-methoxyltryptophan.

Previous studies have confirmed the beneficial role of tryptophan derivatives and AhR ligand in the pathogenesis or treatment of many skin diseases, including inflammatory diseases, skin pigmentation disorders and cancer (38–41, 213). Therefore, we speculate that exogenous supplementation of tryptophan derivatives (such as FICZ, IAId), or targeted intervention of GM (such as *Lactobacillus reuteri*, *Lactobacillus salivary*, *Bifidobacterium* and *Lactobacillus*), or targeted induction of the Kyn-IDO pathway to accelerate endogenous tryptophan metabolism and produce AhR ligands, may ultimately alleviate clinical symptoms of AD by suppressing abnormal immune responses. In addition to being a potential therapeutic option, tryptophan metabolites such as 5-hydroxyindolepyruvate and 5-hydroxyindoleacetic acid may become the new biomarkers supporting the diagnosis and prognosis of atopic diseases. Furthermore, supplementing tryptophan derivatives may alleviate some side effects such as herpes infections and headaches (214, 215) during the treatment of AD with biological agents. However, the biological functions involved in tryptophan metabolism are complex. The outcome of AhR activation depends on the type of cell and ligand (216, 217), stimulation or inhibition of AhR in the skin results in different immune responses (218), such as inducing overexpression of proinflammatory cytokines and ROS production to arise the development of inflammatory diseases or carcinogenesis (219), or affecting the differentiation of Treg cells, thereby promoting the immune tolerance (220, 221). Moreover, there is still insufficient evidence to support the clinical application of tryptophan and its derivatives, and the long-term efficacy and safety of their therapeutic potential are not fully understood. In summary, while the causal relationship among tryptophan metabolism, AD and GM, as well as the specific molecular mechanisms remain to be determined, we have preliminarily provided evidences that tryptophan plays an anti-inflammatory role in AD, laying the foundation for the exciting connection between tryptophan metabolism and AD immune regulation (**Figure 2**). We hope that this review will lead the way to further understand AD, and provide new insights into the pathogenesis and treatment direction of this disease.

## Author contributions

YH: Writing – original draft, Visualization. LC: Writing – original draft. FL: Writing – original draft, Visualization. XX: Writing – review & editing. YO: Writing – review & editing. YD: Visualization, Writing – review & editing.

## References

[1] Wallach D Taïeb A. Atopic dermatitis/atopic eczema. Chem Immunol Allergy (2014) 100:81–96. doi: 10.1159/000358606 24925387

[2] Hidaka T Ogawa E Kobayashi EH Suzuki T Funayama R Nagashima T. The aryl hydrocarbon receptor AhR links atopic dermatitis and air pollution via induction of the neurotrophic factor artemin. Nat Immunol (2017) 18(1):64–73. doi: 10.1038/ni.3614 27869817

[3] Langan SM Irvine AD Weidinger S. Atopic dermatitis. Lancet (2020) 396(10247):345–60. doi: 10.1016/S0140-6736(20)31286-1 32738956

[4] Ricci G Patrizi A Baldi E Menna G Tabanelli M Masi M. Long-term follow-up of atopic dermatitis: retrospective analysis of related risk factors and association with concomitant allergic diseases. J Am Acad Dermatol (2006) 55(5):765–71. doi: 10.1016/j.jaad.2006.04.064 17052480

[5] Kwon MS Lim SK Jang JY Lee J Park HK Kim N. Lactobacillus Sakei WIKIM30 ameliorates atopic dermatitis-like skin lesions by inducing regulatory T cells and altering gut microbiota structure in mice. Front Immunol (2018) 9:1905. doi: 10.3389/fimmu.2018.01905 30154801 PMC6102352

[6] Brandt EB Sivaprasad U. Th2 cytokines and atopic dermatitis. J Clin Cell Immunol (2011) 2:110. doi: 10.4172/2155-9899.1000110 21994899 PMC3189506

[7] Albanesi C Fairchild HR Madonna S Scarponi C De Pità O Leung DY. IL-4 and IL-13 negatively regulate TNF and IFN induced -defensin expression through STAT-6, suppressor of cytokine signaling (SOCS)-1, and SOCS-3. J Immunol (2007) 179:984–92. doi: 10.4049/jimmunol.179.2.984 17617590

[8] Sehra S Yao Y Howell MD Nguyen ET Kansas GS Leung DY. IL-4 regulates skin homeostasis and the predisposition toward allergic skin inflammation. J Immunol (2010) 184:3186–90. doi: 10.4049/jimmunol.0901860 PMC2837507 20147633

[9] Ong PY Ohtake T Brandt C Strickland I Boguniewicz M Ganz T. Endogenous antimicrobial peptides and skin infections in atopic dermatitis. N Engl J Med (2002) 347:1151–60. doi: 10.1056/NEJMoa021481 12374875

[10] Tsakok T Woolf R Smith CH Weidinger S Flohr C. Atopic dermatitis: the skin barrier and beyond. Br J Dermatol (2018) 180(3):464–74. doi: 10.1111/bjd.16934 29969827

[11] Peng W Novak N. Pathogenesis of atopic dermatitis. Clin Exp Allergy (2015) 45:566–74. doi: 10.1111/cea.12495 25610977

[12] Paus R Theoharides TC Arck PC. Neuro-immunoendocrine circuitry of the ‘Brain-skin connection’. Trends Immunol (2006) 27(1):32–9. doi: 10.1016/j.it.2005.10.002 16269267

[13] Yew YW Thyssen JP Silverberg JI. A systematic review and meta-analysis of the regional and age-related differences in atopic dermatitis clinical characteristics. J Am Acad Derm (2019) 80:390–401. doi: 10.1016/j.jaad.2018.09.035 30287309

[14] Bieber T. Atopic dermatitis: An expanding therapeutic pipeline for a complex disease. Nat Rev Drug Discov (2021) 21:21–40. doi: 10.1038/s41573-021-00266-6 34417579 PMC8377708

[15] Eichenfield LF Tom WL Berger TG Krol A Paller AS Schwarzenberger K. Guidelines of care for the management of atopic dermatitis: Section 2. Management and treatment of atopic dermatitis with topical therapies. J Am Acad Dermatol (2014) 71:116–32. doi: 10.1016/j.jaad.2014.03.023 PMC4326095 24813302

[16] Nusbaum KB Nguyen CM Fleischer AB. Emerging systemic therapies for atopic dermatitis: biologics. J Dermatol Treat (2022) 33(3):1269–73. doi: 10.1080/09546634.2020.1836314 33045848

[17] Bao L Zhang H Chan LS. The involvement of the JAK-STAT signaling pathway in chronic inflammatory skin disease atopic dermatitis. JAKSTAT (2013) 2:e24137. doi: 10.4161/jkst.24137 24069552 PMC3772104

[18] Rodrigues MA Torres T. JAK/STAT inhibitors for the treatment of atopic dermatitis. J Dermatolog Treat (2020) 31:33–40. doi: 10.1080/09546634.2019.1577549 30703333

[19] Agus A Planchais J Sokol H. Gut microbiota regulation of tryptophan metabolism in health and disease. Cell Host Microbe (2018) 23(6):716–24. doi: 10.1016/j.chom.2018.05.003 29902437

[20] Taleb S. Tryptophan dietary impacts gut barrier and metabolic diseases. Front Immunol (2019) 10:2113. doi: 10.3389/fimmu.2019.02113 31552046 PMC6746884

[21] Gutierrez-Vazquez C Quintana FJ. Regulation of the immune response by the aryl hydrocarbon receptor. Immunity (2018) 48(1):19–33. doi: 10.1016/j.immuni.2017.12.012 29343438 PMC5777317

[22] Zelante T Iannitti RG Cunha C De Luca A Giovannini G Pieraccini G. Tryptophan catabolites from microbiota engage aryl hydrocarbon receptor and balance mucosal reactivity via interleukin-22. Immunity (2013) 39:372–85. doi: 10.1016/j.immuni.2013.08.003 23973224

[23] Lamas B Richard ML Leducq V Pham HP Michel ML Da Costa G. CARD9 impacts colitis by altering gut microbiota metabolism of tryptophan into aryl hydrocarbon receptor ligands. Nat Med (2016) 22:598–605. doi: 10.1038/nm.4102 27158904 PMC5087285

[24] Dong F Hao F Murray IA Smith PB Koo I Tindall AM. Intestinal microbiota-derived tryptophan metabolites are predictive of Ah receptor activity. Gut Microbes (2020) 12:1–24. doi: 10.1080/19490976.2020.1788899 PMC7524359 32783770

[25] Esser C Rannug A. The aryl hydrocarbon receptor in barrier organ physiology, immunology, and toxicology. Pharmacol Rev (2015) 67:259–79. doi: 10.1124/pr.114.009001 25657351

[26] Lamas B Natividad JM Sokol H. Aryl hydrocarbon receptor and intestinal immunity. Mucosal Immunol (2018) 11(4):1024–38. doi: 10.1038/s41385-018-0019-2 29626198

[27] Gao J Xu K Liu H Liu G Bai M Peng C. Impact of the gut microbiotaon intestinal immunity mediated by tryptophan metabolism. Front Cell Infect Microbiol (2018) 8:13. doi: 10.3389/fcimb.2018.00013 29468141 PMC5808205

[28] Fritsche E Schäfer C Calles C Bernsmann T Bernshausen T Wurm M. Lightening up the UV response by identification of the aryl hydrocarbon receptor as a cytoplasmatic target for ultraviolet B radiation. Proc Natl Acad Sci USA (2007) 104:8851–6. doi: 10.1073/pnas.0701764104 PMC1885591 17502624

[29] Furue M Uchi H Mitoma C Hashimoto-Hachiya A Chiba T Ito T. Antioxidants for healthy skin: The emerging role of aryl hydrocarbon receptors and nuclear factor-erythroid 2-related factor-2. Nutrients (2017) 9:223. doi: 10.3390/nu9030223 28273792 PMC5372886

[30] Magiatis P Pappas P Gaitanis G Mexia N Melliou E Galanou M. Malassezia yeasts produce a collection of exceptionally potent activators of the Ah (dioxin) receptor detected in diseased human skin. J Investig Dermatol (2013) 133:2023–30. doi: 10.1038/jid.2013.92 PMC3714356 23448877

[31] Takei K Mitoma C Hashimoto-Hachiya A Takahara M Tsuji G Nakahara T. Galactomyces fermentation filtrate prevents T helper 2-mediated reduction of filaggrin in an aryl hydrocarbon receptor-dependent manner. Clin Exp Dermatol (2015) 40:786–93. doi: 10.1111/ced.12635 25786502

[32] Furue M Hashimoto-Hachiya A Tsuji G. Antioxidative phytochemicals accelerate epidermal terminal differentiation via the AHR-OVOL1 pathway: Implications for atopic dermatitis. Acta Derm Venereol (2018) 98:918–23. doi: 10.2340/00015555-3003 29972223

[33] Lin YK Leu YL Yang SH Chen HW Wang CT Pang JH. Anti-psoriatic effects of indigo naturalis on the proliferation and differentiation of keratinocytes with indirubin as the active component. J Dermatol Sci (2009) 54:168–74. doi: 10.1016/j.jdermsci.2009.02.007 19303259

[34] Mimura J Ema M Sogawa K Fujii-Kuriyama Y. Identification of a novel mechanism of regulation of Ah (dioxin) receptor function. Genes Dev (1999) 13(1):20–2. doi: 10.1101/gad.13.1.20 PMC316371 9887096

[35] Tsuji G Hashimoto-Hachiya A Kiyomatsu-Oda M Takemura M Ohno F Ito T. Aryl hydrocarbon receptor activation restores filaggrin expression via OVOL1 in atopic dermatitis. Cell Death Dis (2017) 8:e2931. doi: 10.1038/cddis.2017.322 28703805 PMC5550867

[36] Furue M Tsuji G Mitoma C Nakahara T Chiba T Morino-Koga S. Gene regulation of filaggrin and other skin barrier proteins via aryl hydrocarbon receptor. J Dermatol Sci (2015) 80:83–8. doi: 10.1016/j.jdermsci.2015.07.011 26276439

[37] Sutter CH Bodreddigari S Campion C Wible RS Sutter TR. 2,3,7,8-Tetrachlorodibenzo-p-dioxin increases the expression of genes in the human epidermal differentiation complex and accelerates epidermal barrier formation. Toxicol Sci (2011) 124:128–37. doi: 10.1093/toxsci/kfr205 PMC3196651 21835898

[38] Wirthgen E Hoeflich A Rebl A Günther J. Kynurenic Acid: The janus-faced role of an immunomodulatory tryptophan metabolite and its link to pathological conditions. Front Immunol (2018) 8:1957. doi: 10.3389/fimmu.2017.01957 29379504 PMC5770815

[39] Kostyuk VA Potapovich AI Lulli D Stancato A De Luca C Pastore S. Modulation of human keratinocyte responses to solar UV by plant polyphenols as a basis for chemoprevention of non-melanoma skin cancers. Curr Med Chem (2013) 20:869–79. doi: 10.2174/0929867311320070003 23210792

[40] Tsuji G Takahara M Uchi H Takeuchi S Mitoma C Moroi Y. M. An environmental contaminant, benzo(a)pyrene, induces oxidative stress-mediated interleukin-8 production in human keratinocytes via the aryl hydrocarbon receptor signaling pathway. J Dermatol Sci (2011) 62:42–9. doi: 10.1016/j.jdermsci.2010.10.017 21316925

[41] Niestroy J Barbara A Herbst K Rode S van Liempt M Roos PH. Single and concerted effects of benzo[a]pyrene and flavonoids on the AhR and Nrf2-pathway in the human colon carcinoma cell line Caco-2. Toxicol In Vitro (2011) 25:671–83. doi: 10.1016/j.tiv.2011.01.008 21256954

[42] Furue M. Regulation of filaggrin, loricrin, and involucrin by IL-4, Il-13, IL-17a, Il-22, AhR, and NRF2: pathogenic implications in atopic dermatitis. Int J Mol Sci (2020) 21(15):5382. doi: 10.3390/ijms21155382 32751111 PMC7432778

[43] Yu J Luo Y Zhu Z Zhou Y Sun L Gao J. A tryptophan metabolite of the skin microbiota attenuates inflammation in patients with atopic dermatitis through the aryl hydrocarbon receptor. J Allergy Clin Immunol (2019) 143(6):2108–19.e12. doi: 10.1016/j.jaci.2018.11.036 30578876

[44] Furue M Iida K Imaji M Nakahara T. Microbiome analysis of forehead skin in patients with atopic dermatitis and healthy subjects: Implication of Staphylococcus and Corynebacterium. J Dermatol (2018) 45:876–7. doi: 10.1111/1346-8138.14486 29971837

[45] Iwamoto K Moriwaki M Miyake R Hide M. Staphylococcus aureus in atopic dermatitis: Strain-specific cell wall proteins and skin immunity. Allergol Int (2019) 68:309–15. doi: 10.1016/j.alit.2019.02.006 30878567

[46] Roager HM Licht TR. Microbial tryptophan catabolites in health and disease. Nat Commun (2018) 9(1):3294. doi: 10.1038/s41467-018-05470-4 30120222 PMC6098093

[47] Sun M Ma N He T Johnston LJ Ma X. Tryptophan (Trp) modulates gut homeostasis via aryl hydrocarbon receptor (AhR), Crit. Rev Food Sci Nutr (2020) 60(10):1760–8. doi: 10.1080/10408398.2019.1598334 30924357

[48] Islam J Sato S Watanabe K Watanabe T Ardiansyah Hirahara K Aoyama Y. Dietary tryptophan alleviates dextran sodium sulfate-induced colitis through aryl hydrocarbon receptor in mice. J Nutr Biochem (2017) 42:43–50. doi: 10.1016/j.jnutbio.2016.12.019 28113104

[49] Wilck N Matus MG Kearney SM Olesen SW Forslund K Bartolomaeus H. Salt-responsive gut commensal modulates T17 axis and disease. Nature (2017) 551(7682):585–9. doi: 10.1038/nature24628 PMC6070150 29143823

[50] Cervantes-Barragan L Chai JN Tianero MD Di Luccia B Ahern PP Merriman J. Induces gut intraepithelial CD4CD8αα T cells. Sci (New York NY) (2017) 357(6353):806–10. doi: 10.1126/science.aah5825 PMC5687812 28775213

[51] Rothhammer V Mascanfroni ID Bunse L Takenaka MC Kenison JE Mayo L. Type I inter-ferons and microbial metabolites of tryptophan modulate astrocyte activity and CNS inflammation via the aryl hydrocarbon receptor. Nat Med (2016) 22:586–97. doi: 10.1038/nm.4106 PMC4899206 27158906

[52] Metghalchi S Ponnuswamy P Simon T Haddad Y Laurans L Cle´ment M. Indole-amine 2,3-dioxygenase fine-tunes immune homeostasis in atherosclerosis and colitis through repression of interleukin-10 production. Cell Metab (2015) 22:460–71. doi: 10.1016/j.cmet.2015.07.004 26235422

[53] Papathoma E Triga M Fouzas S Dimitriou G. Cesarean section delivery and development of food allergy and atopic dermatitis in early childhood. Pediatr Allergy Immunol (2016) 27(4):419–24. doi: 10.1111/pai.12552 26888069

[54] Brough HA Liu AH Sicherer S Makinson K Douiri A Brown SJ. Atopic dermatitis increases the effect of exposure to peanut antigen in dust on peanut sensitization and likely peanut allergy. J Allergy Clin Immunol (2015) 135(1):164–70. doi: 10.1016/j.jaci.2014.10.007 PMC4282723 25457149

[55] Crestani E Harb H Charbonnier L-M Leirer J Motsinger-Reif A Rachid R. Untargeted metabolomic profiling identifies disease-specific signatures in food allergy and asthma. J Allergy Clin Immunol (2020) 145:897–906. doi: 10.1016/j.jaci.2019.10.014 31669435 PMC7062570

[56] Buyuktiryaki B Sahiner UM Girgin G Birben E Soyer OU Cavkaytar O. Low indoleamine 2,3-dioxygenase activity in persistent food allergy in children. Allergy (2016) 71:258–66. doi: 10.1111/all.12785 26449488

[57] Raitala A Karjalainen J Oja SS Kosunen TU Hurme M. Indoleamine 2,3-dioxygenase (IDO) activity is lower in atopic than in non-atopic individuals and is enhanced by environmental factors protecting from atopy. Mol Immunol (2006) 43:1054–6. doi: 10.1016/j.molimm.2005.06.022 15992929

[58] Murray IA Perdew GH. Ligand activation of the Ah receptor contributes to gastrointestinal homeostasis. Curr Opin Toxicol (2017) 2:15–23. doi: 10.1016/j.cotox.2017.01.003 28944314 PMC5604257

[59] Schulz VJ Smit JJ Willemsen KJ Fiechter D Hassing I Bleumink R. Activation of the aryl hydrocarbon receptor suppresses sensitization in a mouse peanut allergy model. Toxicol Sci (2011) 123:491–500. doi: 10.1093/toxsci/kfr175 21804081

[60] Mims JW. Epidemiology of allergic rhinitis. Int Forum Allergy Rhinol (2014) 4:S18–20. doi: 10.1002/alr.21385 25182349

[61] Aït-Khaled N Pearce N Anderson HR Ellwood P Montefort S Shah J. Global map of the prevalence of symptoms of rhinoconjunctivitis in children: The International Study of Asthma and Allergies in Childhood (ISAAC) Phase Three. Allergy (2009) 64:123–48. doi: 10.1111/j.1398-9995.2008.01884.x 19132975

[62] Loftus PA Wise SK. Epidemiology and economic burden of asthma. Int Forum Allergy Rhinol (2015) 5:S7–S10. doi: 10.1002/alr.21547 26010063

[63] Tao JL Chen YZ Dai QG Tian M Wang SC Shan JJ. Urine metabolic profiles in paediatric asthma. Respirology (2019) 24:572–81. doi: 10.1111/resp.13479 30763984

[64] Saude EJ Skappak CD Regush S Cook K Ben-Zvi A Becker A. Metabolomic profiling of asthma: Diagnostic utility of urine nuclear magnetic resonance spectroscopy. J Allergy Clin Immunol (2011) 127:757–64. doi: 10.1016/j.jaci.2010.12.1077 21377043

[65] Checkley W Deza MP Klawitter J Romero KM Klawitter J Pollard SL. Identifying biomarkers for asthma diagnosis using targeted metabolomics approaches. Respir Med (2016) 121:59–66. doi: 10.1016/j.rmed.2016.10.011 27888993 PMC5516646

[66] Huang Y Chen G Liu X Shao Y Gao P Xin C. Serum metabolomics study and eicosanoid analysis of childhood atopic dermatitis based on liquid chromatography–mass spectrometry. J Proteome Res (2014) 13:5715–23. doi: 10.1021/pr5007069 25316199

[67] Gostner JM Becker K Kofler H Strasser B Fuchs D. Tryptophan metabolism in allergic disorders. Int Arch Allergy Immunol (2016) 169:203–15. doi: 10.1159/000445500 PMC5433561 27161289

[68] Ciprandi G De Amici M Tosca M Fuchs D. Tryptophan metabolism in allergic rhinitis: the effect of pollen allergen exposure. Hum Immunol (2010) 71:911–5. doi: 10.1016/j.humimm.2010.05.017 20540982

[69] Favennec M Hennart B Caiazzo R Leloire A Yengo L Verbanck M. The kynurenine pathway is activated in human obesity and shifted toward kynurenine monooxygenase activation. Obes (Silver Spring) (2015) 23:2066–74. doi: 10.1002/oby.21199 26347385

[70] Romani L Zelante T De Luca A Fallarino F Puccetti P. IL-17 and therapeutic Kynurenines in pathogenic inflammation to fungi. J Immunol (2008) 180(8):5157–62. doi: 10.4049/jimmunol.180.8.5157 18390695

[71] de Araújo EF Feriotti C Galdino NAL Preite NW Calich VLG Loures FV. The IDO–AhR axis controls Th17/Treg immunity in a pulmonary model of fungal infection. Front Immunol (2017) 24:880. doi: 10.3389/fimmu.2017.00880 PMC5523665 28791025

[72] Prendergast GC Malachowski WP DuHadaway JB Muller AJ. Discovery of IDO1 inhibitors: from bench to bedside. Cancer Res (2017) 77(24):6795–811. doi: 10.1158/0008-5472.CAN-17-2285 PMC6021761 29247038

[73] Hossain FMA Park SO Kim HJ Eo JC Choi JY Tanveer M. Indoleamine 2,3-dioxygenase in hematopoietic stem cell-derived cells suppresses rhinovirus-induced neutrophilic airway inflammation by regulating Th1- and Th17-type responses. Immune Netw (2021) 21(4):e26. doi: 10.4110/in.2021.21.e26 34522439 PMC8410990

[74] Hayashi T Beck L Rossetto C Gong X Takikawa O Takabayashi K. Inhibition of experimental asthma by indoleamine 2,3-dioxygenase. J Clin Investig (2004) 114:270–9. doi: 10.1172/JCI21275 PMC449749 15254594

[75] Xu T Zhou Y Qiu L Do DC Zhao Y Cui Z. Aryl hydrocarbon receptor protects lungs from cockroach allergen–induced inflammation by modulating mesenchymal stem cells. J Immunol (2015) 195:5539–50. doi: 10.4049/jimmunol.1501198 PMC4670807 26561548

[76] Li XM Peng J Gu W Guo XJ. TCDD-induced activation of aryl hydrocarbon receptor inhibits Th17 polarization and regulates non-eosinophilic airway inflammation in asthma. PloS One (2016) 11:e0150551. doi: 10.1371/journal.pone.0150551 26938767 PMC4777447

[77] Kepert I Fonseca J Müller C Milger K Hochwind K Kostric M. D-tryptophan from probiotic bacteria influences the gut microbiome and allergic airway disease. J Allergy Clin Immunol (2017) 139:1525–35. doi: 10.1016/j.jaci.2016.09.003 27670239

[78] Hayashi T Mo JH Gong X Rossetto C Jang A Beck L. 3-Hydroxyanthranilic acid inhibits PDK1 activation and suppresses experimental asthma by inducing T cell apoptosis. Proc Natl Acad Sci USA (2007) 104(47):18619–24. doi: 10.1073/pnas.0709261104 PMC2141826 18003900

[79] Papamichael MM Katsardis C Erbas B Itsiopoulos C Tsoukalas D. Urinary organic acids as biomarkers in the assessment of pulmonary function in children with asthma. Nutr Res (2019) 61:31–40. doi: 10.1016/j.nutres.2018.10.004 30683437

[80] Hamilton JD Suarez-Farinas M Dhingra N Cardinale I Li X Kostic A. Dupilumab improves the molecular signature in skin of patients with moderate-to-severe atopic dermatitis. J Allergy Clin Immunol (2014) 134:1293–1300. doi: 10.1016/j.jaci.2014.10.013 25482871

[81] Wu GD Lewis JD. Analysis of the human gut microbiome and association with disease. Clin Gastroenterol Hepatol (2013) 11:774–7. doi: 10.1016/j.cgh.2013.03.038 PMC3822013 23643636

[82] Gilbert JA Blaser MJ Caporaso JG Jansson JK Lynch SV Knight R. Current understanding of the human microbiome. Nat Med (2018) 24(4):392–400. doi: 10.1038/nm.4517 29634682 PMC7043356

[83] Renz H Brandtzaeg P Hornef M. The impact of perinatal immune development on mucosal homeostasis and chronic inflammation. Nat Rev Immunol (2011) 12(1):9–23. doi: 10.1038/nri3112 22158411

[84] Stokes JH Pillsbury DH. The effect on the skin of emotional and nervous states: theoretical and practical consideration of a gastro-intestinal mechanism. Arch Derm Syphilol (1930) 22:962–93. doi: 10.1001/archderm.1930.01440180008002

[85] Salem I Ramser A Isham N Ghannoum MA. The gut microbiome as a major regulator of the gut-skin axis. Front Microbiol (2018) 9:1459. doi: 10.3389/fmicb.2018.01459 30042740 PMC6048199

[86] Stefanovic N Flohr C Irvine AD. The exposome in atopic dermatitis. Allergy (2020) 75(1):63–74. doi: 10.1111/all.13946 31194890 PMC7003958

[87] Stokholm J Blaser MJ Thorsen J Rasmussen MA Waage J Vinding RK. Maturation of the gut microbiome and risk of asthma in childhood. Nat Commun (2018) 9(1):141. doi: 10.1038/s41467-017-02573-2 29321519 PMC5762761

[88] Kim JE Kim HS. Microbiome of the skin and gut in atopic dermatitis (AD): understanding the pathophysiology and finding novel management strategies. J Clin Med (2019) 8(4):444. doi: 10.3390/jcm8040444 30987008 PMC6518061

[89] Lopez-Santamarina A Gonzalez EG Lamas A Mondragon ADC Regal P Miranda JM. Probiotics as a possible strategy for the prevention and treatment of allergies. A narrative review. Foods (2021) 10(4):701. doi: 10.3390/foods10040701 33806092 PMC8064452

[90] Zachariassen LF Krych L Engkilde K Nielsen DS Kot W Hansen CH. Sensitivity to oxazolone induced dermatitis is transferable with gut microbiota in mice. Sci Rep (2017) 7:44385. doi: 10.1038/srep44385 28290517 PMC5349591

[91] Lee SY Lee E Park YM Hong SJ. Microbiome in the gut-skin axis in atopic dermatitis. Allergy Asthma Immunol Res (2018) 10(4):354. doi: 10.4168/aair.2018.10.4.354 29949831 PMC6021588

[92] Song H Yoo Y Hwang J Na YC Kim HS. Faecalibacterium prausnitzii subspecies-level dysbiosis in the human gut microbiome underlying atopic dermatitis. J Allergy Clin Immunol (2016) 137:852–60. doi: 10.1016/j.jaci.2015.08.021 26431583

[93] Wang H Li Y Feng X Li Y Wang W Qiu C. Dysfunctional gut microbiota and relative co-abundance network in infantile eczema. Gut Pathog (2016) 8:36. doi: 10.1186/s13099-016-0118-0 27453732 PMC4957860

[94] Lee MJ Kang MJ Lee SY Lee E Kim K Won S. Perturbations of gut microbiome genes in infants with atopic dermatitis according to feeding type. J Allergy Clin Immunol (2018) 141:1310–9. doi: 10.1016/j.jaci.2017.11.045 29339259

[95] Cait A Cardenas E Dimitriu PA Amenyogbe N Dai D Cait J. Reduced genetic potential for butyrate fermentation in the gut microbiome of infants who develop allergic sensitization. J Allergy Clin Immunol (2019) 144:1638–1647.e3. doi: 10.1016/j.jaci.2019.06.029 31279007

[96] Nylund L Nermes M Isolauri E Salminen S de Vos WM Satokari R. Severity of atopic disease inversely correlates with intestinal microbiota diversity and butyrate-producing bacteria. Allergy (2015) 70:241–4. doi: 10.1111/all.12549 25413686

[97] Zimmermann P Messina N Mohn WW Finlay BB Curtis N. Association between the intestinal microbiota and allergic sensitization, eczema, and asthma: A systematic review. J Allergy Clin Immunol (2019) 143:467–85. doi: 10.1016/j.jaci.2018.09.025 30600099

[98] Ye S Yan F Wang H Mo X Liu J Zhang Y. Diversity analysis of gut microbiota between healthy controls and those with atopic dermatitis in a Chinese population. J Dermatol (2021) 48:158–67. doi: 10.1111/1346-8138.15530 32860635

[99] Hu C van Meel ER Medina-Gomez C Kraaij R Barroso M Kiefte-de Jong J. A population-based study on associations of stool microbiota with atopic diseases in schoolage children. J Allergy Clin Immunol (2021) 148:612–20. doi: 10.1016/j.jaci.2021.04.001 33862008

[100] Melli LCFL do Carmo-Rodrigues MS Araújo-Filho HB Mello CS Tahan S Pignatari ACC. Gut microbiota of children with atopic dermatitis: Controlled study in the metropolitan region of São Paulo, Brazil. Allergol Immunopathol (2020) 48:107–15. doi: 10.1016/j.aller.2019.08.004 32061427

[101] van Nimwegen FA Penders J Stobberingh EE Postma DS Koppelman GH Kerkhof M. Mode and place of delivery, gastrointestinal microbiota, and their influence on asthma and atopy. J Allergy Clin Immunol (2011) 128:948–955.e3. doi: 10.1016/j.jaci.2011.07.027 21872915

[102] Penders J Gerhold K Stobberingh EE Thijs C Zimmermann K Lau S. Establishment of the intestinal microbiota and its role for atopic dermatitis in early childhood. J Allergy Clin Immunol (2013) 132:601–607.e8. doi: 10.1016/j.jaci.2013.05.043 23900058

[103] Zheng H Liang H Wang Y Miao M Shi T Yang F. Altered gut microbiota composition associated with eczema in infants. PloS One (2016) 11:e0166026. doi: 10.1371/journal.pone.0166026 27812181 PMC5094743

[104] Lee E Lee SY Kang MJ Kim K Won S Kim BJ. Clostridia in the gut and onset of atopic dermatitis via eosinophilic inflammation. Ann Allergy Asthma Immunol (2016) 117:91–92.e1. doi: 10.1016/j.anai.2016.04.019 27179583

[105] Kalliomäki M Kirjavainen P Eerola E Kero P Salminen S Isolauri E. Distinct patterns of neonatal gut microflora in infants in whom atopy was and was not developing. J Allergy Clin Immunol (2001) 107:129–34. doi: 10.1067/mai.2001.111237 11150002

[106] Watanabe S Narisawa Y Arase S Okamatsu H Ikenaga T Tajiri Y. Differences in fecal microflora between patients with atopic dermatitis and healthy control subjects. J Allergy Clin Immunol (2003) 111:587–91. doi: 10.1067/mai.2003.105 12642841

[107] Mah KW Björkstén B Lee BW van Bever HP Shek LP Tan TN. Distinct pattern of commensal gut microbiota in toddlers with eczema. Int Arch Allergy Immunol (2006) 140:157–63. doi: 10.1159/000092555 16601353

[108] Hong PY Lee BW Aw M Shek LPC Yap GC Chua KY. Comparative analysis of fecal microbiota in infants with and without eczema. PloS One (2010) 5:e9964. doi: 10.1371/journal.pone.0009964 20376357 PMC2848600

[109] Roduit C Frei R Ferstl R Loeliger S Westermann P Rhyner C. High levels of butyrate and propionate in early life are associated with protection against atopy. Allergy (2019) 74(4):799–809. doi: 10.1111/all.13660 30390309

[110] Kim HJ Lee SH Hong SJ. Antibiotics-induced dysbiosis of intestinal microbiota aggravates atopic dermatitis in mice by altered short-chain fatty acids. Allergy Asthma Immunol Res (2020) 12(1):137–48. doi: 10.4168/aair.2020.12.1.137 PMC6875482 31743970

[111] Tan J McKenzie C Potamitis M Thorburn AN Mackay CR Macia L. The role of short-chain fatty acids in health and disease. Adv Immunol (2014) 121:91–119. doi: 10.1016/B978-0-12-800100-4.00003-9 24388214

[112] Macia L Tan J Vieira AT Leach K Stanley D Luong S. Metabolite-sensing receptors GPR43 and GPR109A facilitate dietary fibre-induced gut homeostasis through regulation of the inflammasome. Nat Commun (2015) 6:6734. doi: 10.1038/ncomms7734 25828455

[113] Arpaia N Campbell C Fan X Dikiy S van der Veeken J deRoos P. Metabolites produced by commensal bacteria promote peripheral regulatory T-cell generation. Nature (2013) 504(7480):451–5. doi: 10.1038/nature12726 PMC3869884 24226773

[114] Furusawa Y Obata Y Fukuda S Endo TA Nakato G Takahashi D. Commensal microbe-derived butyrate induces the differentiation of colonic regulatory T cells. Nature (2013) 504(7480):446–50. doi: 10.1038/nature12721 24226770

[115] Kendrick SFW O’Boyle G Mann J Zeybel M Palmer J Jones DE. Acetate, for key modulator of inflammatory responses in acute alcoholic hepatitis. Hepatology (2010) 51:1988–97. doi: 10.1002/hep.23572 20232292

[116] Usami M Kishimoto K Ohata A Miyoshi M Aoyama M Fueda Y. Butyrate and trichostatin A attenuate nuclear factor kappaB activation and tumor necrosis factor alpha secretion and increase prostaglandin E2 secretion in human peripheral blood mononuclear cells. Nutr Res (2008) 28:321–8. doi: 10.1016/j.nutres.2008.02.012 19083427

[117] Kim M Qie Y Park J Kim CH. Gut microbial metabolites fuel host antibody responses. Cell Host Microbe (2016) 20:202–14. doi: 10.1016/j.chom.2016.07.001 PMC4982788 27476413

[118] Jeong DY Ryu MS Yang HJ Jeong SY Zhang T Yang HJ. Pediococcus acidilactici intake decreases the clinical severity ofAtopic dermatitis along with increasing mucin production and improving the gut microbiome in NC/Nga mice. BioMed Pharmacother (2020) 129:110488. doi: 10.1016/j.biopha.2020.110488 32768968

[119] Kandikattu HK Upparahalli Venkateshaiah S Mishra A. Synergy of interleukin (IL)-5 and IL-18 in eosinophil mediated pathogenesis of allergic diseases. Cytokine Growth Factor Rev (2019) 47:83–98. doi: 10.1016/j.cytogfr.2019.05.003 31126874 PMC6781864

[120] Goodarzi A Mozafarpoor S Bodaghabadi M Mohamadi M. The potential of probiotics for treating acne vulgaris: A review of literature on acne and microbiota. Dermatol Ther (2020) 33(3):e13279. doi: 10.1111/dth.13279 32266790

[121] Yu Y Dunaway S Champer J Kim J Alikhan A. Changing our microbiome: probiotics in dermatology. Br J Dermatol (2020) 182(1):39–46. doi: 10.1111/bjd.18088 31049923

[122] Liu Y Tian X Daniel RC Okeugo B Armbrister SA Luo M. Impact of probiotic Limosilactobacillus reuteri DSM 17938 on amino acid metabolism in the healthy newborn mouse. Amino Acids (2022) 54(10):1383–401. doi: 10.1007/s00726-022-03165-1 PMC9643610 35536363

[123] Xia J Jiang S Lv L Wu W Wang Q Xu Q. Modulation of the immune response and metabolism in germ-free rats colonized by the probiotic Lactobacillus salivarius LI01. Appl Microbiol Biotechnol (2021) 105(4):1629–45. doi: 10.1007/s00253-021-11099-z 33507355

[124] Wiedlocha M Marcinowicz P Janoska-Jazdzik M Szulc A. Gut microbiota, Kynurenine pathway and mental disorders - Review. Prog Neuropsychopharmacol Biol Psychiatry (2021) 106:110145. doi: 10.1016/j.pnpbp.2020.110145 33203568

[125] Marsland BJ. Regulating inflammation with microbial metabolites. Nat Med (2016) 22(6):581–3. doi: 10.1038/nm.4117 27270775

[126] Wikoff WR Anfora AT Liu J Schultz PG Lesley SA Peters EC. Metabolomics analysis reveals large effects of gut microflora on mammalian blood metabolites. Proc Natl Acad Sci USA (2009) 106:3698–703. doi: 10.1073/pnas.0812874106 19234110 PMC2656143

[127] Matsumoto M Ebata T Hirooka J Hosoya R Inoue N Itami S. Antipruritic effects of the probiotic strain LKM512 in adults with atopic dermatitis. Ann Allergy Asthma Immunol (2014) 113(2):209–216.e7. doi: 10.1016/j.anai.2014.05.002 24893766

[128] Macpherson AJ Slack E Geuking MB McCoy KD. The mucosal firewalls against commensal intestinal microbes. Semin Immunopathol (2009) 31(2):145–9. doi: 10.1007/s00281-009-0174-3 19707762

[129] Purchiaroni F Tortora A Gabrielli M Bertucci F Gigante G Ianiro G. The role of intestinal microbiota and the immune system. Eur Rev Med Pharmacol Sci (2013) 17:323–33. 23426535

[130] Seite S Bieber T. Barrier function and microbiotic dysbiosis in atopic dermatitis. Clin Cosmet Investig Dermatol (2015) 8:479–83. doi: 10.2147/CCID.S91521 PMC4576901 26396539

[131] Johnson CC Ownby DR. The infant gut bacterial microbiota and risk of pediatric asthma and allergic diseases. Trans Res (2017) 179:60–70. doi: 10.1016/j.trsl.2016.06.010 PMC5555614 27469270

[132] Bansal T Alaniz RC Wood TK Jayaraman A. The bacterial signal indole increases epithelial-cell tight-junction resistance and attenuates indicators of inflammation. Proc Natl Acad Sci USA (2010) 107(1):228–33. doi: 10.1073/pnas.0906112107 PMC2806735 19966295

[133] Shimada Y Kinoshita M Harada K Mizutani M Masahata K Kayama H. Commensal bacteria-dependent indole production enhances epithelial barrier function in the colon. PloS One (2013) 8(11):e80604. doi: 10.1371/journal.pone.0080604 24278294 PMC3835565

[134] Wlodarska M Luo C Kolde R d’Hennezel E Annand JW Heim CE. Indoleacrylic acid produced by commensal peptostreptococcus species suppresses inflammation. Cell Host Microbe (2017) 22(1):25–37 e26. doi: 10.1016/j.chom.2017.06.007 28704649 PMC5672633

[135] Jennis M Cavanaugh CR Leo GC Mabus JR Lenhard J Hornby PJ. Microbiota-derived tryptophan indoles increase after gastric bypass surgery and reduce intestinal permeability *in vitro* and *in vivo* . Neurogastroenterol Motil (2018) 30(2):1–12. doi: 10.1111/nmo.13178 28782205

[136] Platten M von Knebel Doeberitz N Oezen I Wick W Ochs K. Cancer immunotherapy by targeting IDO1/TDO and their downstream effectors. Front Immunol (2015) 5:673. doi: 10.3389/fimmu.2014.00673 25628622 PMC4290671

[137] Ma Q. Influence of light on aryl hydrocarbon receptor signaling and consequences in drug metabolism, physiology and disease. Expert Opin Drug Metab Toxicol (2011) 7:1267–93. doi: 10.1517/17425255.2011.614947 21883026

[138] Chieosilapatham P Kiatsurayanon C Umehara Y Trujillo-Paez JV Peng G Yue H. Keratinocytes: innate immune cells in atopic dermatitis. Clin Exp Immunol (2021) 204(3):296–309. doi: 10.1111/cei.13575 33460469 PMC8119845

[139] Mitamura Y Nunomura S Nanri Y Ogawa M Yoshihara T Masuoka M. The IL-13/periostin/IL-24 pathway causes epidermal barrier dysfunction in allergic skin inflammation. Allergy (2018) 73(9):1881–91. doi: 10.1111/all.13437 29528494

[140] Tsuji G Ito T Chiba T Mitoma C Nakahara T Uchi H. The role of the OVOL1-OVOL2 axis in normal and diseased human skin. J Dermatol Sci (2018) 90:227–31. doi: 10.1016/j.jdermsci.2018.02.005 29454536

[141] Takemura M Nakahara T Hashimoto-Hachiya A Furue M Tsuji G. Glyteer, soybean tar, impairs IL-4/Stat6 signaling in murine bone marrow-derived dendritic cells: The basis of its therapeutic effect on atopic dermatitis. Int J Mol Sci (2018) 19:1169. doi: 10.3390/ijms19041169 29649105 PMC5979322

[142] Di Meglio P Duarte JH Ahlfors H Owens ND Li Y Villanova F. Activation of the aryl hydrocarbon receptor dampens the severity of inflammatory skin conditions. Immunity (2014) 40:989–1001. doi: 10.1016/j.immuni.2014.04.019 24909886 PMC4067745

[143] Mosser DM Edwards JP. Exploring the full spectrum of macrophage activation. Nat Rev Immunol (2008) 8(12):958–69. doi: 10.1038/nri2448 PMC2724991 19029990

[144] Wang XF Wang HS Wang H Zhang F Wang KF Guo Q. The role of indoleamine 2,3-dioxygenase (IDO) in immune tolerance: focus on macrophage polarization of THP-1 cells. Cell Immunol (2014) 289:42–8. doi: 10.1016/j.cellimm.2014.02.005 24721110

[145] Savage NDL de Boer T Walburg KV Joosten SA van Meijgaarden K Geluk A. Human anti-inflammatory macrophages induce Foxp3 + GIT + CD25 + regulatory T cells, which suppress via membrane-bound TGFb-1. J Immunol (2008) 181:2220–6. doi: 10.4049/jimmunol.181.3.2220 18641362

[146] Wang YF Hsu YJ Wu HF Lee GL Yang YS Wu JY. Endothelium-derived 5-methoxytryptophan is a circulating anti-inflammatory molecule that blocks systemic inflammation. Circ Res (2016) 119:222–36. doi: 10.1161/CIRCRESAHA.116.308559 27151398

[147] Tiszlavicz Z Németh B Fülöp F Vécsei L Tápai K Ocsovszky I. Different inhibitory effects of Kynurenic acid and a novel Kynurenic acid analogue on tumour necrosis factor-a (TNF-a) production by mononuclear cells, HMGB1 production by monocytes and HNP1-3 secretion by neutrophils. Naunyn Schmiedebergs Arch Pharmacol (2011) 383:447–55. doi: 10.1007/s00210-011-0605-2 21336543

[148] Yoshida Y Hayakawa K Fujishiro M Ikeda K Tsushima H Hirai T. Social defeat stress exacerbates atopic dermatitis through downregulation of DNA methyltransferase 1 and upregulation of C-C motif chemokine receptor 7 in skin dendritic cells. Biochem Biophys Res Commun (2020) 529(4):1073–9. doi: 10.1016/j.bbrc.2020.06.157 32819567

[149] Grohmann U Bianchi R Belladonna ML Silla S Fallarino F Fioretti MC. IFN-gamma inhibits presentation of a tumor/self peptide by CD8 alpha-dendritic cells via potentiation of the CD8+ subset. J Immunol (2000) 165:1357–63. doi: 10.4049/jimmunol.165.3.1357 10903738

[150] Hill M Tanguy-Royer S Royer P Chauveau C Asghar K Tesson L. IDO expands human CD4 +CD25high regulatory T cells by promoting maturation of LPS-treated dendritic cells. Eur J Immunol (2007) 37:3054–62. doi: 10.1002/eji.200636704 17948274

[151] Fallarino F Grohmng KW Hwang KW Orabona C Vacca C Bianchi R. Modulation of tryptophan catabolism by regulatory T cells. Nat Immunol (2003) 4:1206–12. doi: 10.1038/ni1003 14578884

[152] Mellor AL Munn DH. IDO expression by dendritic cells: tolerance and tryptophan catabolism. Nat Rev Immunol (2004) 4:762–74. doi: 10.1038/nri1457 15459668

[153] Terness P Chuang JJ Opelz G. The immunoregulatory role of IDO-producing human dendritic cells revisited. Trends Immunol (2006) 27:68–73. doi: 10.1016/j.it.2005.12.006 16406698

[154] Aoki R Aoki-Yoshida A Suzuki C Takayama Y. Indole-3-pyruvic acid, an aryl hydrocarbon receptor activator, suppresses experimental colitis in mice. J Immunol (2018) 201(12):3683–93. doi: 10.4049/jimmunol.1701734 30429284

[155] Ständer S. Atopic dermatitis. N Engl J Med (2021) 384(12):1136–43. doi: 10.1056/NEJMra2023911 33761208

[156] Lee JK Seok JK Cho I Yang G Kim KB Kwack SJ. Topical application of celastrol alleviates atopic dermatitis symptoms mediated through the regulation of thymic stromal lymphopoietin and group 2 innate lymphoid cells. J Toxicol Environ Health A (2021) 84(22):922–31. doi: 10.1080/15287394.2021.1955785 34304725

[157] Kolodkin-Gal I Romero D Cao S Clardy J Kolter R Losick R. D-amino acids trigger biofilm disassembly. Science (2010) 328(5978):627–9. doi: 10.1126/science.1188628 PMC2921573 20431016

[158] Kao WT Frye M Gagnon P Vogel JP Chole R. D-amino acids do not inhibit pseudomonas aeruginosa biofilm formation. Laryngoscope Investig Otolaryngol (2017) 2(1):4–9. doi: 10.1002/lio2.34 PMC5324625 28286870

[159] Leung DY. New insights into atopic dermatitis: Role of skin barrier and immune dysregulation. Allergol Int (2013) 62:151–61. doi: 10.2332/allergolint.13-RAI-0564 PMC8609663 23712284

[160] Huang YJ Marsland BJ Bunyavanich S O’Mahony L Leung DY Muraro A. The microbiome in allergic disease: Current understanding and future opportunities—2017 PRACTALL document of the American Academy of Allergy, Asthma & Immunology and the European Academy of Allergy and Clinical Immunology. J Allergy Clin Immunol (2017) 139:1099–110. doi: 10.1016/j.jaci.2017.02.007 PMC5899886 28257972

[161] Kiyomatsu-Oda M Uchi H Morino-Koga S Furue M. Protective role of 6-formylindolo[3,2-b] carbazole (FICZ), an endogenous ligand for arylhydrocarbon receptor, in chronic mite-induced dermatitis. J Dermatol Sci (2018) 90:284–94. doi: 10.1016/j.jdermsci.2018.02.014 29500077

[162] Hong CH Lee CH Yu HS Huang SK. Benzopyrene, a major polyaromatic hydrocarbon in smoke fume, mobilizes Langerhans cells and polarizes Th2/17 responses in epicutaneous protein sensitization through the aryl hydrocarbon receptor. Int Immunopharmacol (2016) 36:111–7. doi: 10.1016/j.intimp.2016.04.017 27129092

[163] Rannug A Fritsche E. The aryl hydrocarbon receptor and light. Biol Chem (2006) 387:1149–57. doi: 10.1515/BC.2006.143 16972782

[164] Morita A. Current developments in phototherapy for psoriasis. J Dermatol (2018) 45:287–92. doi: 10.1111/1346-8138.14213 29369396

[165] Ortiz-Salvador JM Pérez-Ferriols A. Phototherapy in atopic dermatitis. Adv Exp Med Biol (2017) 996:279–86. doi: 10.1007/978-3-319-56017-5_23 29124708

[166] Kennedy LH Sutter CH Leon Carrion S Tran QT Bodreddigari S Kensicki E. 2,3,7,8-Tetrachlorodibenzo-p-dioxin-mediated production of reactive oxygen species is an essential step in the mechanism of action to accelerate human keratinocyte differentiation. Toxicol Sci (2013) 132:235–49. doi: 10.1093/toxsci/kfs325 PMC3576006 23152189

[167] Edamitsu T Taguchi K Kobayashi EH Okuyama R Yamamoto M. Aryl hydrocarbon receptor directly regulates artemin gene expression. Mol Cell Biol (2019) 39(20):e00190−19. doi: 10.1128/MCB.00190-19 31358547 PMC6766698

[168] Lou H Lu J Choi EB Oh MH Jeong M Barmettler S. Expression of IL-22 in the skin causes Th2-biased immunity, epidermal barrier dysfunction, and pruritus via stimulating epithelial Th2 cytokines and the GRP pathway. J Immunol (2017) 198(7):2543–55. doi: 10.4049/jimmunol.1600126 PMC5360537 28228560

[169] Singh RK Lee KM Vujkovic−Cvijin I Ucmak D Farahnik B Abrouk M. The role of IL−17 in vitiligo: A review. Autoimmun Rev (2016) 15:397−404. doi: 10.1016/j.autrev.2016.01.004 26804758 PMC4769658

[170] Mattapallil MJ Kielczewski JL Zárate−Bladés CR St Leger AJ Raychaudhuri K Silver PB. Interleukin 22 ameliorates neuropathology and protects from central nervous system autoimmunity. J Autoimmun (2019) 102:65−76. doi: 10.1016/j.jaut.2019.04.017 31080013 PMC6667188

[171] Neil JA Matsuzawa−Ishimoto Y Kernbauer−Hölzl E Schuster SL Sota S Venzon M. IFN−I and IL−22 mediate protective effects of intestinal viral infection. Nat Microbiol (2019) 4:1737−1749. doi: 10.1038/s41564-019-0470-1 31182797 PMC6871771

[172] Zenewicz LA. IL−22: There is a gap in our knowledge. Immunohorizons (2018) 2:198−207. doi: 10.4049/immunohorizons.1800006 31022687 PMC10518850

[173] Hou Q Ye L Liu H Huang L Yang Q Turner JR. Lactobacillus accelerates ISCs regeneration to protect the integrity of intestinal mucosa through activation of STAT3 signaling pathway induced by LPLs secretion of IL-22. Cell Death Differ (2018) 25(9):1657–70. doi: 10.1038/s41418-018-0070-2 PMC6143595 29459771

[174] Park H Li Z Yang XO Chang SH Nurieva R Wang YH. A distinct lineage of CD4 T cells regulates tissue inflammation by producing interleukin 17[J/OL]. Nat Immunol (2005) 6(11):1133–41. doi: 10.1038/ni1261 PMC1618871 16200068

[175] Hofmann MA Fluhr JW Ruwwe-Glösenkamp C Stevanovic K Bergmann KC Zuberbier T. Role of IL-17 in atopy-A systematic review. Clin Trans Allergy (2021) 11(6):e12047. doi: 10.1002/clt2.12047 PMC8361814 34429872

[176] Liu T Li S Ying S Tang S Ding Y Li Y. The IL-23/IL-17 pathway in inflammatory skin diseases: from bench to bedside. Front Immunol (2020) 11:594735. doi: 10.3389/fimmu.2020.594735 33281823 PMC7705238

[177] Hvid M Vestergaard C Kemp K Christensen GB Deleuran B Deleuran M. IL-25 in atopic dermatitis: A possible link between inflammation and skin barrier dysfunction? J Invest Dermatol (2011) 131(1):150–7. doi: 10.1038/jid.2010.277 20861853

[178] Sugaya M. The role of Th17-related cytokines in atopic dermatitis. Int J Mol Sci (2020) 21(4):1314. doi: 10.3390/ijms21041314 32075269 PMC7072946

[179] Rothhammer V Mascanfroni ID Bunse L Takenaka MC Kenison JE Mayo L. Type I interferons and microbial metabolites of tryptophan modulate astrocyte activity and central nervous system inflammation via the aryl hydrocarbon receptor. Nat Med (2016) 22:586–97. doi: 10.1038/nm.4106 PMC4899206 27158906

[180] Monteleone I Rizzo A Sarra M Sica G Sileri P Biancone L. Aryl hydrocarbon receptor-induced signals up-regulate IL-22 production and inhibit inflammation in the gastrointestinal tract. Gastroenterology (2011) 141(1):237–248, 48 e1. doi: 10.1053/j.gastro.2011.04.007 21600206

[181] Singh NP Singh UP Singh B Price RL Nagarkatti M Nagarkatti PS. Activation of aryl hydrocarbon receptor (AhR) leads to reciprocal epigenetic regulation of FoxP3 and IL-17 expression and amelioration of experimental colitis. PloS One (2011) 6(8):e23522. doi: 10.1371/journal.pone.0023522 21858153 PMC3156147

[182] Huang Z Jiang Y Yang Y Shao J Sun X Chen J. 3,3′-Diindolylmethane alleviates oxazolone-induced colitis through Th2/Th17 suppression and Treg induction. Mol Immunol (2013) 53(4):335–44. doi: 10.1016/j.molimm.2012.09.007 23085552

[183] Rohlman D Pham D Yu Z Steppan LB Kerkvliet NI. Aryl hydrocarbon receptor−Mediated perturbations in gene expression during early stages of CD4(+) T−cell differentiation. Front Immunol (2012) 3:223. doi: 10.3389/fimmu.2012.00223 22888330 PMC3412388

[184] Opitz CA Litzenburger UM Sahm F Ott M Tritschler I Trump S. An endogenous tumour-promoting ligand of the human aryl hydrocarbon receptor. Nature (2011) 478:197–203. doi: 10.1038/nature10491 21976023

[185] Mezrich JD Fechner JH Zhang X Johnson BP Burlingham WJ Bradfield CA. An interaction between Kynurenine and the aryl hydrocarbon receptor can generate regulatory T cells. J Immunol (2010) 185:3190–8. doi: 10.4049/jimmunol.0903670 PMC2952546 20720200

[186] Wu D Molofsky AB Liang HE Ricardo-Gonzalez RR Jouihan HA Bando JK. Eosinophils sustain adipose alternatively activated macrophages associated with glucose homeostasis. Science (2011) 332:243–7. doi: 10.1126/science.1201475 PMC3144160 21436399

[187] Beamer CA Seaver BP Shepherd DM. Aryl hydrocarbon receptor (AhR) regulates silica-induced inflammation but not fibrosis. Toxicol Sci (2012) 126:554–68. doi: 10.1093/toxsci/kfs024 PMC3307612 22273745

[188] Frumento G Rotondo R Tonetti M Damonte G Benatti U Ferrara GB. Tryptophan-derived catabolites are responsible for inhibition of T and natural killer cell proliferation induced by indoleamine 2,3-dioxygenase. J Exp Med (2002) 196:459–68. doi: 10.1084/jem.20020121 PMC2196046 12186838

[189] Fallarino F Grohmann U Vacca C Bianchi R Orabona C Spreca A. T cell apoptosis by tryptophan catabolism. Cell Death Differ (2002) 9:1069–77. doi: 10.1038/sj.cdd.4401073 12232795

[190] Orabona C Puccetti P Vacca C Bicciato S Luchini A Fallarino F. et al, Toward the identification of a tolerogenic signature in IDO-competent dendritic cells. Blood (2006) 107:2846–54. doi: 10.1182/blood-2005-10-4077 16339401

[191] Neavin DR Liu D Ray B Weinshilboum RM. The role of the aryl hydrocarbon receptor (AhR) in immune and inflammatory diseases. Int J Mol Sci (2018) 19(12):3851. doi: 10.3390/ijms19123851 30513921 PMC6321643

[192] Kaiser H Parker E Hamrick MW. Hamrick, Kynurenine signaling through the aryl hydrocarbon receptor: implications for aging and healthspan. Exp Gerontol (2020) 130:110797. doi: 10.1016/j.exger.2019.110797 31786316 PMC7899131

[193] Lanis JM Alexeev EE Curtis VF Kitzenberg DA Kao DJ Battista KD. Tryptophan metabolite activation of the aryl hydrocarbon receptor regulates IL-10 receptor expression on intestinal epithelia. Mucosal Immunol (2017) 10(5):1133–44. doi: 10.1038/mi.2016.133 PMC5515702 28098246

[194] Beutelspacher SC Tan PH McClure MO Larkin DF Lechler RI George AJ. Expression of indoleamine 2,3-dioxygenase (IDO) by endothelial cells: implications for the control of alloresponses. Am J Transpl (2006) 6:1320–30. doi: 10.1111/j.1600-6143.2006.01324.x 16686756

[195] Odemuyiwa SO Ghahary A Li Y Puttagunta L Lee JE Musat-Marcu S. Cutting edge: human eosinophils regulate T cell subset selection through indoleamine 2,3-dioxygenase. J Immunol (2004) 173:5909–13. doi: 10.4049/jimmunol.173.10.5909 15528322

[196] Munn DH Mellor AL. IDO and tolerance to tumors. Trends Mol Med (2004) 10:15–8. doi: 10.1016/j.molmed.2003.11.003 14720581

[197] Uyttenhove C Pilotte L Theate I Stroobant V Colau D Parmentier N. Evidence for a tumoral immune resistance mechanism based on tryptophan degradation by indoleamine 2,3-dioxygenase. Nat Med (2003) 9:1269–74. doi: 10.1038/nm934 14502282

[198] Agaugue S Perrin-Cocon L Coutant F Andre P Lotteau V. 1-Methyl-tryptophan can interfere with TLR signaling in dendritic cells independently of IDO activity. J Immunol (2006) 177:2061–71. doi: 10.4049/jimmunol.177.4.2061 PMC2377404 16887964

[199] Munn DH. Indoleamine 2,3-dioxygenase, tumor-induced tolerance and counter-regulation. Curr Opin Immunol (2006) 18:220–5. doi: 10.1016/j.coi.2006.01.002 16460921

[200] Puccetti P Fallarino F. Generation of T cell regulatory activity by plasmacytoid dendritic cells and tryptophan catabolism. Blood Cells Mol Dis (2008) 40:101–5. doi: 10.1016/j.bcmd.2007.06.026 17881253

[201] Mellor AL Munn DH. Tryptophan catabolism and regulation of adaptive mmunity. J Immunol (2003) 170(12):5809–13. doi: 10.4049/jimmunol.170.12.5809 12794104

[202] Pallotta MT Orabona C Volpi C Vacca C Belladonna ML Bianchi R. Indoleamine 2,3-dioxygenase is a signaling protein in long-term tolerance by dendritic cells. Nat Immunol (2011) 12:870–8. doi: 10.1038/ni.2077 21804557

[203] Sharma MD Baban B Chandler P Hou DY Singh N Yagita H. Plasmacytoid dendritic cells from mouse tumor-draining lymph nodes directly activate mature Tregs via indoleamine 2,3- dioxygenase. J Clin Invest (2007) 117:2570–82. doi: 10.1172/JCI31911 PMC1940240 17710230

[204] Munn DH Sharma MD Baban B Harding HP Zhang Y Ron D. GCN2 kinase in T cells mediates proliferative arrest and anergy induction in response to indoleamine 2,3-dioxygenase. Immunity (2005) 22:633–42. doi: 10.1016/j.immuni.2005.03.013 15894280

[205] Munn DH Mellor AL. Indoleamine 2,3 dioxygenase and metabolic control of immune responses. Trends Immunol (2013) 34:137–43. doi: 10.1016/j.it.2012.10.001 PMC3594632 23103127

[206] Curti A Pandolfi S Valzasina B Aluigi M Isidori A Ferri E. Modulation of tryptophan catabolism by human leukemic cells results in the conversion of CD25– into CD25+ T regulatory cells. Blood (2007) 109:2871–7. doi: 10.1182/blood-2006-07-036863 17164341

[207] Xu H Zhang GX Ciric B Rostami A. IDO: a double-edged sword for T(H)1/T(H)2 regulation. Immunol Lett (2008) 121:1–6. doi: 10.1016/j.imlet.2008.08.008 18824197 PMC2628165

[208] Odemuyiwa SO Ghahary A Li Y Puttagunta L Lee JE Musat-Marcu S. Cutting edge: human eosinophils regulate T cell subset selection through indoleamine 2,3-dioxygenase. J Immunol (2004) 173:5909–13. doi: 10.4049/jimmunol.173.10.5909 15528322

[209] Molano A Illarionov PA Besra GS Putterman C Porcelli SA. Modulation of invariant natural killer T cell cytokine responses by indoleamine 2,3-dioxygenase. Immunol Lett (2008) 117:81–90. doi: 10.1016/j.imlet.2007.12.013 18272236 PMC2367367

[210] Musso T Gusella G Brooks A Longo D Varesio L. Interleukin-4 inhibits indoleamine 2,3-dioxygenase expression in human monocytes. Blood (1994) 83:1408–11. doi: 10.1182/blood.v83.5.1408.1408 8118042

[211] Chaves AC Cerávolo IP Gomes JA Zani CL Romanha AJ Gazzinelli RT. IL-4 and IL-13 regulate the induction of indoleamine 2,3-dioxygenase activity and the control of Toxoplasma gondii replication in human fibroblasts activated with IFN-gamma. Eur J Immunol (2001) 31:333–44. doi: 10.1002/1521-4141(200102)31:2<333::AID-IMMU333>3.0.CO;2-X 11180096

[212] Ito M Ogawa K Takeuchi K Nakada A Heishi M Suto H. Gene expression of enzymes for tryptophan degradation pathway is upregulated in the skin lesions of patients with atopic dermatitis or psoriasis. J Derm Sci (2004) 36:157–64. doi: 10.1016/j.jdermsci.2004.08.012 15541637

[213] Hu T Pan Z Yu Q Mo X Song N Yan M. Benzo(a)pyrene induces interleukin (IL)-6 production and reduces lipid synthesis in human SZ95 sebocytes via the aryl hydrocarbon receptor signaling pathway. Environ Toxicol Pharmacol (2016) 43:54–60. doi: 10.1016/j.etap.2016 26963242

[214] Adams O Besken K Oberdörfer C MacKenzie CR Rüssing D Däubener W. Inhibition of human herpes simplex virus type 2 by interferon gamma and tumor necrosis factor alpha is mediated by indoleamine 2,3-dioxygenase. Microbes Infect (2004) 6(9):806–12. doi: 10.1016/j.micinf.2004.04.007 15374002

[215] Drummond PD. Tryptophan depletion increases nausea, headache and photophobia in migraine sufferers. Cephalalgia (2006) 26(10):1225–33. doi: 10.1111/j.1468-2982.2006.01212.x 16961791

[216] Wincent E Bengtsson J Bardbori AM Alsberg T Luecke S Rannug U. A. Inhibition of cytochrome P4501-dependent clearance of the endogenous agonist FICZ as a mechanism for activation of the aryl hydrocarbon receptor. Proc Natl Acad Sci USA (2012) 109:4479–84. doi: 10.1073/pnas.1118467109 PMC3311358 22392998

[217] Sun YV Boverhof DR Burgoon LD Fielden MR Zacharewski TR. Comparative analysis of dioxin response elements in human, mouse and rat genomic sequences. Nucleic Acids Res (2004) 32:4512–23. doi: 10.1093/nar/gkh782 PMC516056 15328365

[218] Haarmann-Stemmann T Abel J Fritsche E Krutmann J. The AhR-Nrf2 pathway in keratinocytes: On the road to chemopre-vention? J Investig Dermatol (2012) 132:7–9. doi: 10.1038/jid.2011.359 22158605

[219] Tsuji G Takahara M Uchi H Matsuda T Chiba T Takeuchi S. Identification of ketoconazole as an AhR-Nrf2 activator in cultured human keratinocytes: The basis of its anti-inflammatory effect. J Investig Dermatol (2012) 132:59–68. doi: 10.1038/jid.2011.194 21753779

[220] di Meglio P Perera GK Nestle FO. The multitasking organ: Recent insights into skin immune function. Immunity (2011) 35:857–69. doi: 10.1016/j.immuni.2011.12.003 22195743

[221] van der Fits L Mourits S Voerman JS Kant M Boon L Laman JD. Imiquimod-induced psoriasis-like skin inflammation in mice is mediated via the IL-23/IL-17 axis. J Immunol (2009) 182:5836–45. doi: 10.4049/jimmunol.0802999 19380832

